# Effects of aging on protein expression in mice brain microvessels: ROS scavengers, mRNA/protein stability, glycolytic enzymes, mitochondrial complexes, and basement membrane components

**DOI:** 10.1007/s11357-021-00468-1

**Published:** 2021-10-28

**Authors:** Partha K. Chandra, Sinisa Cikic, Ibolya Rutkai, Jessie J. Guidry, Prasad V. G. Katakam, Ricardo Mostany, David W. Busija

**Affiliations:** 1 Department of Pharmacology, Tulane University School of Medicine, 1430 Tulane Avenue #8683, New Orleans, LA 70112 USA; 2 Tulane Brain Institute, Tulane University, 200 Flower Hall, New Orleans, LA USA; 3 Department of Biochemistry and Molecular Biology, Louisiana State University Health Science Center, New Orleans, LA 70112 USA; 4 Proteomics Core Facility, Louisiana State University Health Science Center, New Orleans, LA 70112 USA

**Keywords:** Cortical microvessels, Proteomics, Brain aging, ROS scavengers, mRNA/protein stability, Glycolytic/mitochondrial proteins

## Abstract

Differentially expressed (DE) proteins in the cortical microvessels (MVs) of young, middle-aged, and old male and female mice were evaluated using discovery-based proteomics analysis (> 4,200 quantified proteins/group). Most DE proteins (> 90%) showed no significant differences between the sexes; however, some significant DE proteins showing sexual differences in MVs decreased from young (8.3%), to middle-aged (3.7%), to old (0.5%) mice. Therefore, we combined male and female data for age-dependent comparisons but noted sex differences for examination. Key proteins involved in the oxidative stress response, mRNA or protein stability, basement membrane (BM) composition, aerobic glycolysis, and mitochondrial function were significantly altered with aging. Relative abundance of superoxide dismutase-1/-2, catalase and thioredoxin were reduced with aging. Proteins participating in either mRNA degradation or pre-mRNA splicing were significantly increased in old mice MVs, whereas protein stabilizing proteins decreased. Glycolytic proteins were not affected in middle age, but the relative abundance of these proteins decreased in MVs of old mice. Although most of the 41 examined proteins composing mitochondrial complexes I–V were reduced in old mice, six of these proteins showed a significant reduction in middle-aged mice, but the relative abundance increased in fourteen proteins. Nidogen, collagen, and laminin family members as well as perlecan showed differing patterns during aging, indicating BM reorganization starting in middle age. We suggest that increased oxidative stress during aging leads to adverse protein profile changes of brain cortical MVs that affect mRNA/protein stability, BM integrity, and ATP synthesis capacity.

**Supplementary Information:**

The online version contains supplementary material available at 10.1007/s11357-021-00468-1.

## Introduction

Cerebral microvessels (MVs: end arterioles, capillaries, and venules) are essential for maintenance of nutrient supply for brain metabolic needs while ensuring immunological and physical sequestration of brain tissues from harmful circulating substances via the blood–brain barrier (BBB) and basement membrane (BM) [1]. MVs are also the most vulnerable-to-stress segments of the brain vasculature. They must maintain high ATP production rates via glycolysis and oxidative phosphorylation (OXPHOS), essential for a variety of functions: the regulation of capillary perfusion pressure, transport across the BBB, BM maintenance, and tight nutrient delivery/brain metabolism coupling. They are also subject to exposure from potentially disruptive or toxic agents released from neurons or circulating blood. Aging is an unavoidable stress with an ever-increasing detrimental effect on the brain microvasculature, which affects neuronal health and function and adds vulnerability to strokes and dementias, such as Alzheimer’s disease (AD). Gross anatomical age-related changes also are reported: decreases in small blood vessel [2] and capillary density [3–5], looping, tortuosity, and twisting [6–11] of MVs, and reorganization and BBB leakage. Unfortunately, the role of different proteins in cortical MVs in the etiology of aging and development of neurological diseases has received little attention due to a prior focus on large arteries and because of methodological challenges in interpreting the complexity of factors involved in the synthesis and stability of proteins and protein interactions.

Recently, we reported on our proteomics approaches to examine expression and interactions of large numbers of proteins in MVs in young male and female rodents, with an emphasis on mitochondrial and related proteins [12, 13]. In the current study, we have expanded our investigation and have performed a more extensive examination of proteins involved in the structure and function of MVs using a discovery-based quantitative proteomics approach quantifying more than 4200 differentially expressed (DE) proteins/group in cortical MVs of young, middle-aged, and old male and female mice. Specifically, we focused on proteins involved in BM formation, ROS scavenging, mRNA/protein stability maintenance, and ATP production via glycolysis and OXPHOS.

## Materials and methods

### Animals

Age-matched, male and female, young (4–6 months), middle-aged (12–14 months), and old (20–21 months) mice were included in this study. Mice were obtained from Jackson Laboratory [Tg(Thy1-EGFP)MJrs/J] (Jax No. 007788) and bred in a C57B16J background. Sample sizes included 3 male and 3 female MVs per age group. Mice were kept in group housing at ~ 23 ºC on a 12-h light/dark cycle with ad libitum access to food and water. This study conforms to the Institutional Animal Care and Use Committee guidelines of Tulane University, the National Institutes of Health Office of Laboratory Animal Welfare guidelines, and the ARRIVE guidelines for animal research. All MVs were collected at the same time of day to avoid any differences due to circadian rhythm.

### Microvessels isolation

The MVs were isolated as previously described [12, 13]. Large, superficial, blood-vessel-free, cortical tissue from mice brains was homogenized in ice cold Dulbecco’s phosphate-buffered saline (DPBS) (Life Technologies Corporation, NY, USA) and centrifuged at 3300 × g for 15 min. The pellet was resuspended in 17.5% dextran (Thermo Fisher Scientific, Waltham, MA) and passed through a 300-µm filter (pluriSelect Life Science, CA, USA). The filtrate was centrifuged at 7900 × g for 15 min. The contaminated myelin was eliminated, and the MV pellet was resuspended in 2% bovine serum albumin (BSA) (Sigma-Aldrich, St Louis, MO) and passed through a 70-µm filter (Corning Incorporated, NY, USA). To achieve impurity-free MVs, the subsequent sample was centrifuged at 13,000 × g for 15 min with a final clean-up with dextran (17.5%) followed by BSA (2%). Last, the MV pellet was resuspended in DPBS and stored at − 80 °C until used. The MV preparation integrity has been validated as described in our studies [12–15].

### Quantitative discovery-based proteomics using tandem mass tags (TMT) and liquid chromatography mass spectrometry (LC–MS)

Samples were prepared for discovery-based quantitative proteomic analysis by the addition of 1% SDS and sonicated until completely homogenous. The protein concentration was determined using BCA protein assay kit (Pierce, Thermo Scientific) and an eight-point standard curve. Based on the protein concentration, 100 µg of each protein sample was prepared for trypsin digestion by reducing the cysteines with tris(2-carboxyethyl)phosphine followed by alkylation with iodoacetamide. After chloroform–methanol precipitation, each protein pellet was digested with 1 µg trypsin overnight at 37 °C. Tryptic peptides were labeled using one of three TMT 6-plex reagents sets (Thermo Scientific Pierce); old, middle, and young.

An equal amount of each TMT-labeled sample was pooled in a single tube with SepPak purified (Waters, Ireland) using acidic reverse phase conditions. We used off-line fractionation to reduce the sample complexity, as previously described [12, 13]. The fractionated, labeled peptide mixtures were run on a Dionex U3000 nano-flow system coupled to a Thermo Fisher Fusion Orbitrap mass spectrometer. Each fraction was subjected to a 95-min chromatographic method employing a gradient from 2 to 25% ACN in 0.1% formic acid (FA) (ACN/FA) over the course of 65 min, a gradient of 50% ACN/FA for an additional 10 min, and then 90% ACN/FA for 5 min, with a 15-min re-equilibration into 2% ACN/FA. Chromatography was carried out in a “trap-and-load” format using an EASY-Spray source (Thermo); trap column C18 PepMap 100, 5 µm, 100 A, and the separation column was an EASY-Spray PepMap RSLC C18 2 µm, 100 A, 75 µm × 25 cm (Thermo Fisher Dionex, Sunnyvale, CA). The entire run had a flow rate of 0.3 µL/min. Electrospray was achieved at 1.8 kV.

We used an MS3 approach for TMT data acquisition, as previously described [16]. Survey scans (MS1) were performed in the Orbitrap using 120,000 resolutions. Data-dependent MS2 scans in the linear ion trap used a collision-induced dissociation (CID) of 25%. Reporter ions were fragmented using a high-energy collision dissociation (HCD) of 55% and detected in the Orbitrap at 50,000 resolutions (MS3). This was repeated for three technical replicates. The 3 runs of each age group were searched using the SEQUEST HT node of Proteome Discoverer 2.4 (Thermo). The Protein FASTA database was the *Mus musculus*, SwissProt tax ID = 10,090, version 2017–10-25 containing 25,097 sequences. Static modifications included TMT reagents on lysine and N-terminus (+ 229.163); carbamidomethyl on cysteines (+ 57.021); dynamic phosphorylation of serine, threonine, and tyrosine (+ 79.966 Da); and dynamic modification of oxidation of methionine (+ 15.9949). Parent ion tolerance was 10 ppm, fragment mass tolerance was 0.6 Da for MS2 scans, and the maximum number of missed cleavages was set to 2.

### Statistical analysis

Only high scoring peptides were considered using a false discovery rate of < 1%, and only one unique high-scoring peptide was required for inclusion of an identified protein in our results. Proteome Discoverer was also used to determine quantitative differences between biological groups. We used a *t* test analysis for quantitative data by grouping biological replicates and performing pair-wise comparisons for fold change: old, middle-aged, and young mice. The normalized abundance quantity of a biological replicate was calculated from an average of three experimental replicates. The data was presented as mean ± standard deviation (SD). Initially, the data sets were assessed by the Shapiro–Wilk test for normality followed by unpaired *t* test with Welch correction for normally distributed data. When the data did not pass the normality test, a non-parametric Mann–Whitney test was performed as indicated in the figure legends. The statistical analysis was performed using GraphPad Prism version 9.0.0 for Windows, and *p* < 0.05 was considered statistically significant.

## Results

### Age- and sex-specific quantification of DE proteins in cortical MVs of mice

More than 4200 DE proteins were quantified in cortical MVs of young, middle-aged, and old mice. The number of significant sex-dependent DE proteins (abundance ratio: female/male) in cortical MVs generally decreased from young (8.3%), middle-aged (3.7%), to old (0.5%) mice (Supplementary Table 1). Due to the notable (> 90%) lack of significant sex differences, especially in old mice MVs, we combined male and female data to strengthen the statistical analyses. When sex differences were more prominent and important to consider, we note this information in the text and include details in Supplementary Materials.

### Oxidative stress response proteins were altered with aging in mice cortical MVs

**Fig. 1:**
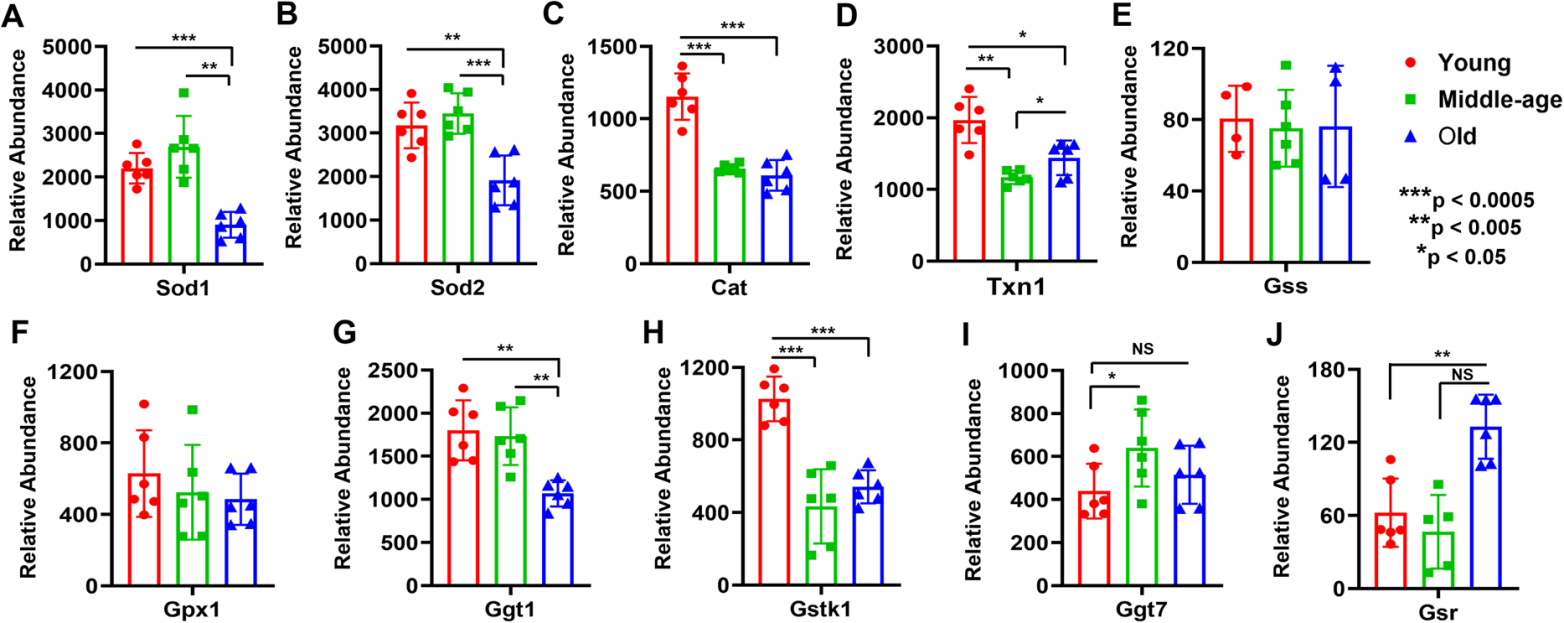
Decreased expression of oxidative stress response proteins with aging in mouse cortical MVs (panels **A**–**J**). Relative protein abundance quantified by TMT-based LC–MS study. Proteins that exhibited group differences are shown as bar graphs with red, green, and blue for young, middle-aged, and old mice, respectively. Graphs show mean ± SD of relative abundance, with between group significant differences indicated by asterisks. NS: not significant. All protein data sets passed the Shapiro–Wilk normality test, and unpaired *t* test with Welch correction. Age-matched, three males and three females were included in each group (*n* = 6/group). Sod1 (SOD1), superoxide dismutase 1; Sod2 (SOD2), superoxide dismutase 2; Cat (CAT), catalase; Txn1 (TXN1), thioredoxin; Gss (GSS), glutathione synthetase; Gpx1 (GPX1), glutathione peroxidase 1; Ggt1 (GGT1), glutathione hydrolase 1 proenzyme; Gstk1 (GSTK1), glutathione S-transferase kappa 1; Ggt7 (GGT7), glutathione hydrolase 7; Gsr (GSR), glutathione reductase

The expressions of superoxide dismutase 1 (SOD1) and superoxide dismutase 2 (SOD2) were significantly decreased in aged compared with young and middle-aged mice (Fig. 1A–B). Moreover, the expressions of catalase (CAT) and thioredoxin (TXN1) were significantly decreased both in middle-aged and old mice MVs (Fig. 1C–D). While glutathione synthase (GSS) and glutathione peroxidase-1 (GPX1) levels were not significantly reduced during aging (Fig. 1E–F), the enzymes involved in the glutathione cycle were significantly differentially expressed in MVs of young, middle-aged, and old mice. For example, glutathione hydrolase 1 proenzyme (GGT1)-expression in old mice MVs was significantly decreased compared with middle-aged or young mice (Fig. 1G). Interestingly, the expression of glutathione S transferase kappa 1 (GSTK1) was significantly decreased, but glutathione hydrolase 7-expression was increased in middle-aged MVs (Fig. 1H–I). Remarkably, glutathione reductase-expression was significantly higher in old compared with young mice MVs (Fig. 1J).

### Proteins involved in mRNA/protein stability changed during aging

**Fig. 2:**
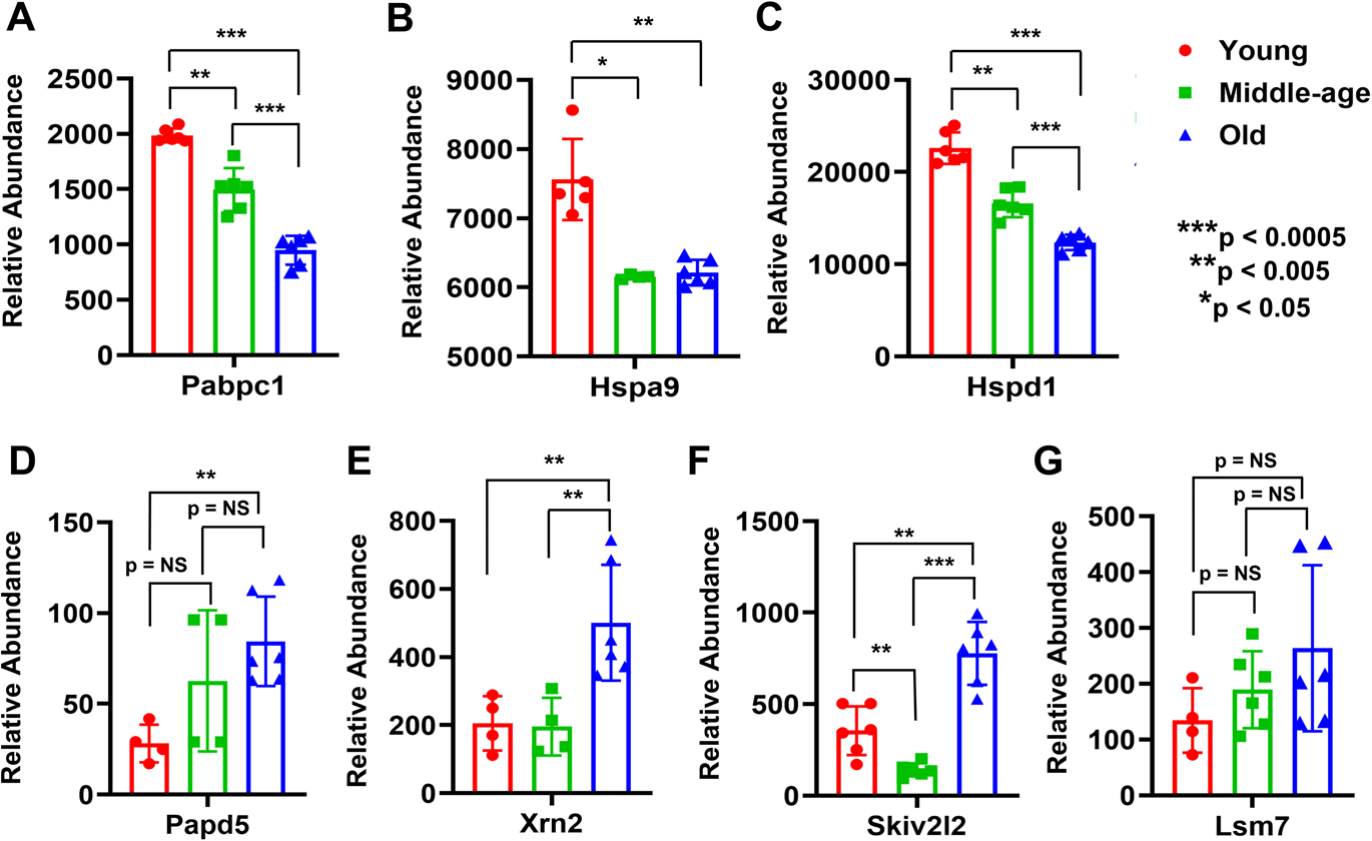
Proteins involved in mRNA/protein stability changed during aging in mice MVs (panels **A**–**G**). Proteins that exhibited between group differences are shown as colored bar graphs. Graphs show mean ± SD of relative abundance, with significant differences between groups presented as indicated. NS: not significant. Data presented in panels passed the Shapiro–Wilk normality test except HSPA9 and PAPD5, which and were followed by an unpaired t test with Welch correction. The non-parametric Mann–Whitney test was used for HSPA9 and PAPD5. Age-matched, three males and three females were included in each group (*n* = 6/group). Pabpc1 (PABPC1), Polyadenylate-binding protein 1; Hspa9 (HSPA9), stress-70 protein, mitochondrial; Hspd1 (HSPD1), 60 kDa heat shock protein, mitochondrial; Papd5 (PAPD5), non-canonical poly(A) RNA polymerase PAPD5; Xrn2 (XRN2), 5′-3′ exoribonuclease 2; Skiv2l2 (SKIV2L2), superkiller viralicidic activity 2-like 2; Lsm7 (LSM7), U6 snRNA-associated Sm-like protein LSM7

Proteins involved in either mRNA stability (polyadenylate-binding protein 1: PABPC1) or proper protein folding (mitochondrial 60 and 70 kDa heat shock proteins: HSPA9 and HSPD1, respectively) were more decreased in middle-aged and old cortical MVs than young MVs (Fig. 2A–C). Alternatively, non-canonical poly(A) RNA polymerase PAPD5 (Papd5), 5′-3′ exoribonuclease 2 (XRN2), and superkiller viralicidic activity 2-like 2 (SKIV2l2) which are involved in either mRNA degradation or pre-mRNA splicing were significantly upregulated in MVs of old compared with middle-aged and young mice (Fig. 2D–F). The expression of U6 snRNA-associated Sm-like protein LSM7 (LSM7), which plays an important role in pre-mRNA splicing via spliceosome, was also increased in middle-aged and old mice MVs (Fig. 2G).

### Glycolytic enzymes were significantly decreased with aging in mice

**Fig. 3:**
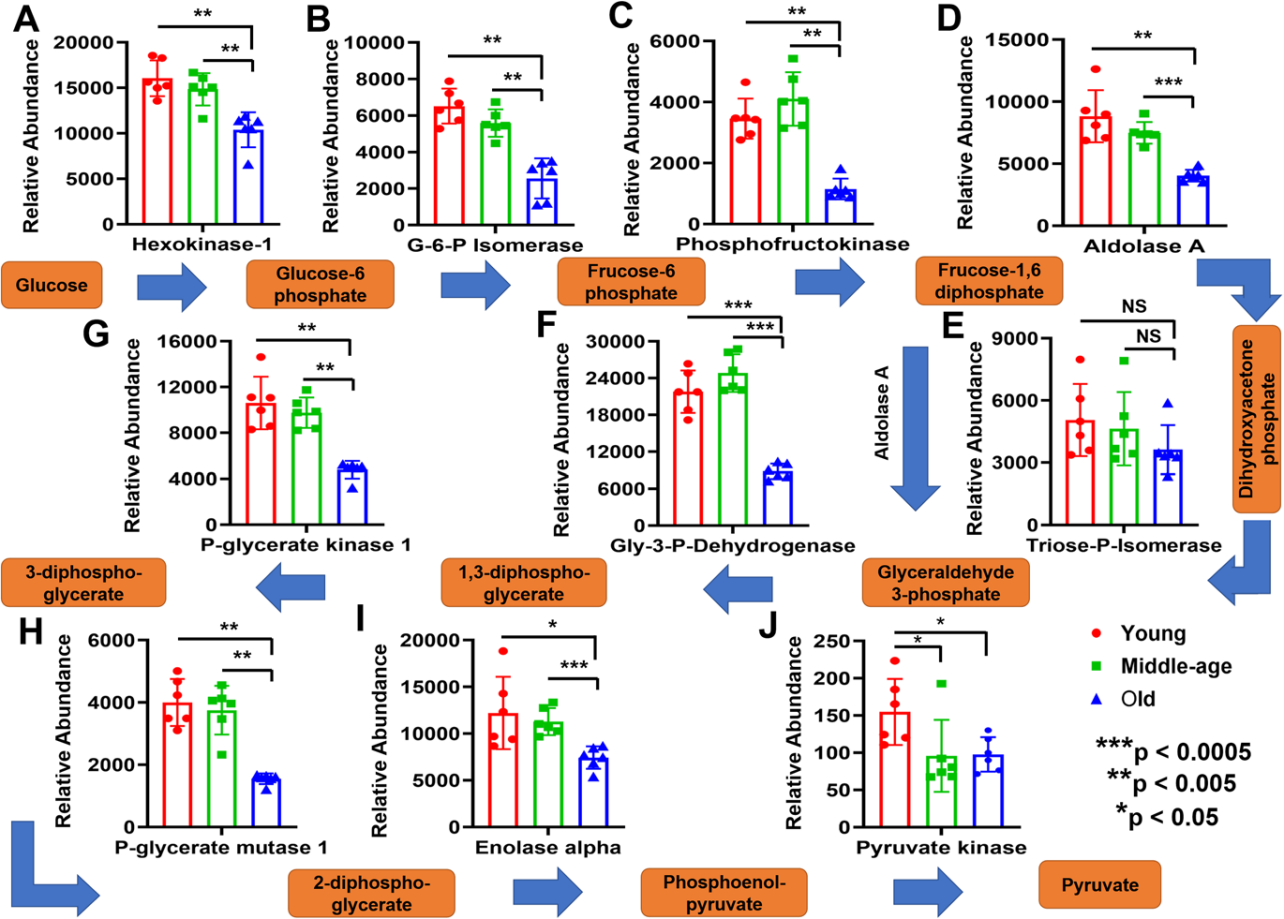
Decreased expression of glycolytic enzymes in mouse cortical MVs with aging (panels **A**–**J**). Proteomics of glycolysis and associated pathways. Stepwise intermediate products are highlighted in brown, and the pathway is indicated by blue arrows. The abundant expression of enzymes involved in each step is presented in panels **A** to **J**. The enzymes that exhibited between group differences are shown as bar graphs with red, green, and blue for young, middle-aged, and old mice, respectively. Graphs show mean ± SD of relative abundance, with significant differences between groups indicated by asterisks. The protein abundance in MVs of old mice is presented in panels (**A**), (**B**), (**C**), (**E**), (**G**), (**H**), and (**J**). These panels did not pass the Shapiro–Wilk normality test, and the non-parametric Mann–Whitney test was used. Proteins in panels (**D**), (**F**), and (**I**) passed the Shapiro–Wilk normality test and were followed by unpaired *t* test with Welch correction. Age-matched, three males and three females were included in each group (*n* = 6/group). G-6-P Isomerase, glucose-6-phosphate isomerase; Triose-P-isomerase, triosephosphate isomerase; Gly-3-p-Dehydogenase, glyceraldehyde 3-phosphate dehydrogenase; P-glycerate kinase 1, phosphoglycerate kinase 1; P-glycerate mutase 1, phosphoglycerate mutase 1

Aging led to reductions in almost all enzyme levels involved in glycolysis in old compared with young and middle-aged mice, including hexokinase-1 (Fig. 3A), glucose-6-phosphate isomerase (Fig. 3B), phosphofructokinase 1 (Fig. 3C), aldolase A (Fig. 3D), glyceraldehyde 3-phosphate dehydrogenase (GAPDH) (Fig. 3F), phosphoglycerate kinase 1 (Fig. 3G), phosphoglycerate mutase 1 (PGAM1) (Fig. 3H), enolase alpha (Fig. 3I), and pyruvate kinase (Fig. 3J). Only triosephosphate isomerase (TPI) (Fig. 3E) was not significantly reduced in old mice. The expression of glycolytic proteins was similar between males and females for all age groups except for GAPDH and PGAM1 in young mice (Supplementary Fig. 1).

### OXPHOS proteins were affected by age

More than 800 mitochondrial and related DE proteins in cortical MVs of young, middle-aged, and old mice were quantified. The number of sex-dependent significant mitochondria-related DE proteins (abundance ratio: female/male) in cortical MVs gradually decreased from young (6.5%: 58/885) to middle-aged (4.4%: 36/816) to old mice (0.7%: 6/830) (Supplementary Table 2). Data point proximity in each graph for complexes I–V support our male and female data grouping; details of sex differences are presented in Supplementary Figures.

**Fig. 4:**
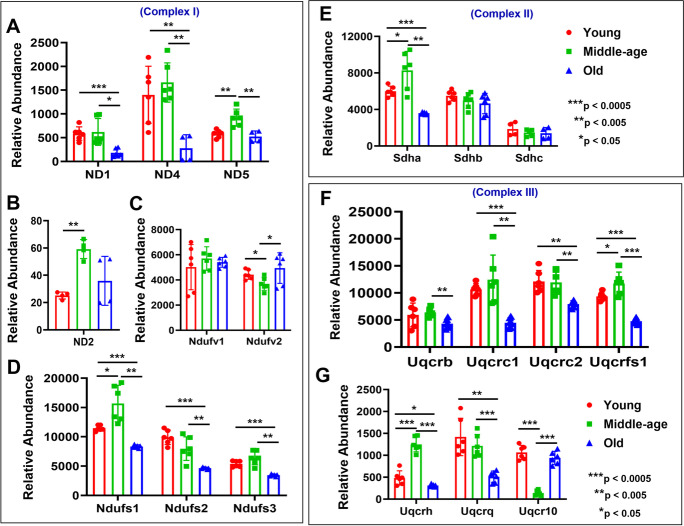
Altered age-specific expression of mitochondrial complexes I, II, and III proteins in mouse cortical MVs (panels **A**–**G**). Abundant expression of different proteins involved in mitochondrial complex I (A–D), complex II (E), and complex III (F–G) that exhibited differences between groups are shown as bar graphs. Graphs show mean ± SD of relative abundance, with significant differences between groups presented as indicated. Proteins presented in different panels passed the Shapiro–Wilk normality test followed by unpaired *t* test with Welch correction. Age-matched, three males and three females were included in each group (*n* = 6/group). ND1, NADH-ubiquinone oxidoreductase chain 1; ND2, NADH-ubiquinone oxidoreductase chain 2; ND4, NADH-ubiquinone oxidoreductase chain 4; ND5, NADH-ubiquinone oxidoreductase chain 5; Ndufv1 (NDUFV1), NADH dehydrogenase [ubiquinone] flavoprotein 1; Ndufv2 (NDUFV2), NADH dehydrogenase [ubiquinone] flavoprotein 2; Ndufs1 (NDUFS1), NADH-ubiquinone oxidoreductase 75 kDa subunit, mitochondrial; Ndufs2 (NDUFS2), NADH dehydrogenase [ubiquinone] iron-sulfur protein 2; Ndufs3 (NDUFS3), NADH dehydrogenase [ubiquinone] iron-sulfur protein 3; Sdha (SDHA), succinate dehydrogenase [ubiquinone] flavoprotein subunit; Sdhb (SDHB), succinate dehydrogenase [ubiquinone] iron-sulfur subunit; Sdhc (SDHC), succinate dehydrogenase cytochrome b560 subunit; Uqcrb (UQCRB), cytochrome b-c1 complex subunit 7; Uqcrc1 (UQCRC1), cytochrome b-c1 complex subunit 1; Uqcrc2 (UQCRC2), cytochrome b-c1 complex subunit 2; Uqcrfs1 (UQCRFS1), cytochrome b-c1 complex subunit Rieske; Uqcrh (UQCRH), cytochrome b-c1 complex subunit 6; Uqcrq (UQCRQ), cytochrome b-c1 complex subunit 8; Uqcr10 (UQCR10), cytochrome b-c1 complex subunit 9

**Fig. 5:**
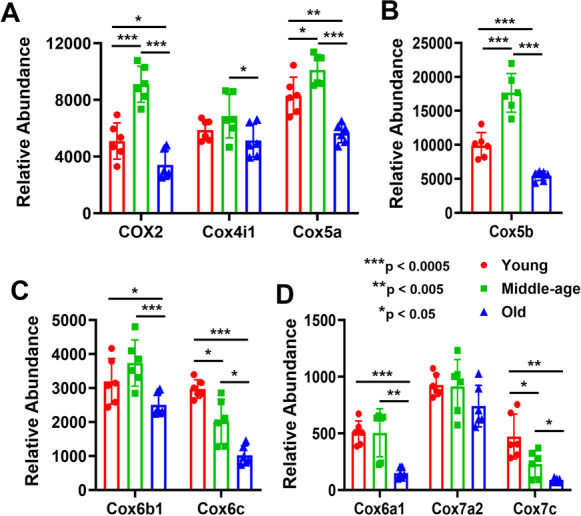
Altered expression of mitochondrial complex IV proteins in mouse cortical MVs during aging (panels **A**–**D**). Proteins involved in mitochondrial complex IV that exhibited between group differences are shown as colored bar graphs as in Figs. 1, 2, 3, and 4. Graphs show mean ± SD of relative abundance, with significant differences between groups presented as indicated. Proteins presented in different panels passed the test for normal distribution and were followed by unpaired *t* test with Welch correction. Age-matched, three males and three females were included in each group (*n* = 6/group). COX2, cytochrome c oxidase subunit 2; Cox4i1 (COX4I1), cytochrome c oxidase subunit 4 isoform 1; Cox5a (COX5A), cytochrome c oxidase subunit 5A; Cox5b (COX5B), cytochrome c oxidase subunit 5B; Cox6a1 (COX6A1), cytochrome c oxidase subunit 6A1; Cox6b1 (COX6B1), cytochrome c oxidase subunit 6B1; Cox6c (COX6C), cytochrome c oxidase subunit 6C; Cox7a2 (COX7A2), cytochrome c oxidase subunit 7A2; Cox7c (COX7C), cytochrome c oxidase subunit 7C

**Fig. 6:**
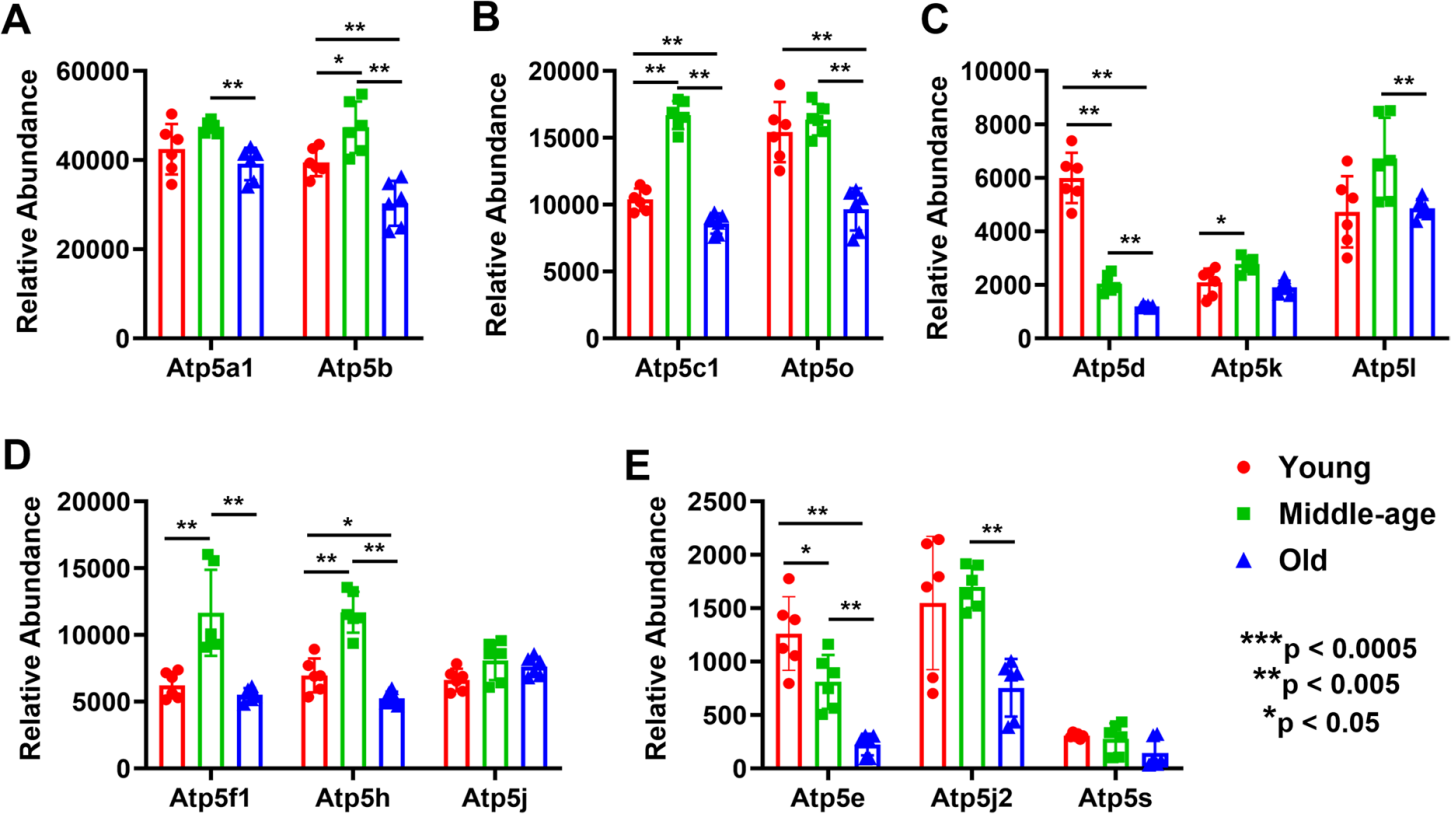
Age-specific expression of proteins in mitochondrial complex V in mouse cortical MVs (panels **A**–**E**). Abundant expression of different proteins that demonstrate between group differences are shown as bar graphs. Graphs show mean ± SD of relative abundance, with significant between group differences indicated by asterisks. Proteins presented in different panels did not pass the Shapiro–Wilk test for normal distribution. The non-parametric Mann–Whitney test was performed for statistical significance in different groups. Age-matched, three males and three females were included in each group (*n* = 6/group). Atp5a1 (ATP5A1), ATP synthase subunit alpha; Atp5b (ATP5B), ATP synthase subunit beta; Atp5c1 (ATP5C1), ATP synthase subunit gamma; Atp5d (ATP5D), ATP synthase subunit delta; Atp5e (ATP5E), ATP synthase subunit epsilon; Atp5f1 (ATP5F1), ATP synthase F(0) complex subunit B1; Atp5h (ATP5H), ATP synthase subunit d; Atp5j (ATP5J), ATP synthase-coupling factor 6; Atp5j2 (ATP5J2), ATP synthase subunit f; Atp5k (ATP5K), ATP synthase subunit e; Atp5l (ATP5L), ATP synthase subunit g; Atp5o (ATP5O), ATP synthase subunit O; Atp5s (ATP5S), ATP synthase subunit s

The overall aging effect shows widespread decreases in old compared with young and middle-aged mice MVs in all five complexes (6/9 for complex I, 1/3 for complex II, 6/7 for complex III, 8/9 for complex IV, and 10/13 for complex V) (Figs. 4, 5, and 6). A surprising aspect of OXPHOS protein expression is that middle-aged mice often showed a significant increase in specific proteins compared with young mice before these decreased in old mice. We see this pattern with ND2 and NDUFS1 in complex I (Fig. 4B and D); SDHA in complex II (Fig. 4E); UQCRFS1 and UQCRH in complex III (Fig. 4F and G); COX2, COX5a, and COX5b in complex IV (Fig. 5A–B); and ATP5B, ATP5C1, ATP5F1, and ATP5H in complex V (Fig. 6A, B, and D). However, in some cases, mitochondrial proteins in MVs decreased in middle age compared with young mice: NDUFV2 in complex I (Fig. 4C), 1/9; none for complex II (Fig. 4); UQCR10 for complex III (Fig. 4G), 1/7; COX6C and COX7C for complex IV (Fig. 5C and D), 2/9; and ATP5D and ATP5E for complex V, 2/13 (Fig. 6C and E). In complex I, the expression of NDUFS2 and NDUFS3 was significantly decreased in young female MVs (Supplementary Fig. 2A), whereas NDUFS1 was significantly decreased in middle-aged female than male (Supplementary Fig. 2B). In complex II, SDHA expression was significantly less only in old female than male (Supplementary Fig. 3C). In complex III, UQCR10 in young female and UQCRC2 and UQCRB in middle-aged female were significantly less expressed than male (Supplementary Fig. 4A–B). Similarly, in complex IV, the expression of COX4I1 and COX6C in young female, COX2 and COX5A in middle-aged female, and COX7C in old female was significantly less than in male (Supplementary Fig. 5A–C). In complex V, the expression of ATP5C1 was significantly decreased in both young and middle-aged female mice. Interestingly, the relative abundance of ATP5J was significantly higher in young female but was decreased significantly in old female MVs (Supplementary Fig. 6A–C).

Although the OXPHOS proteins largely show parity between the sexes, the expression of other mitochondrial-related proteins shows a more complex pattern (data not presented). For example, proteins involved in mitochondrial transcription/translation are heavily slanted to males in young mice, and proteins involved in fatty acid metabolism are more abundantly expressed in female compared with male mice in middle age. The other mitochondrial-related proteins are equally expressed in male and females in old mice.

### Basement membrane proteins were affected by age

**Fig. 7:**
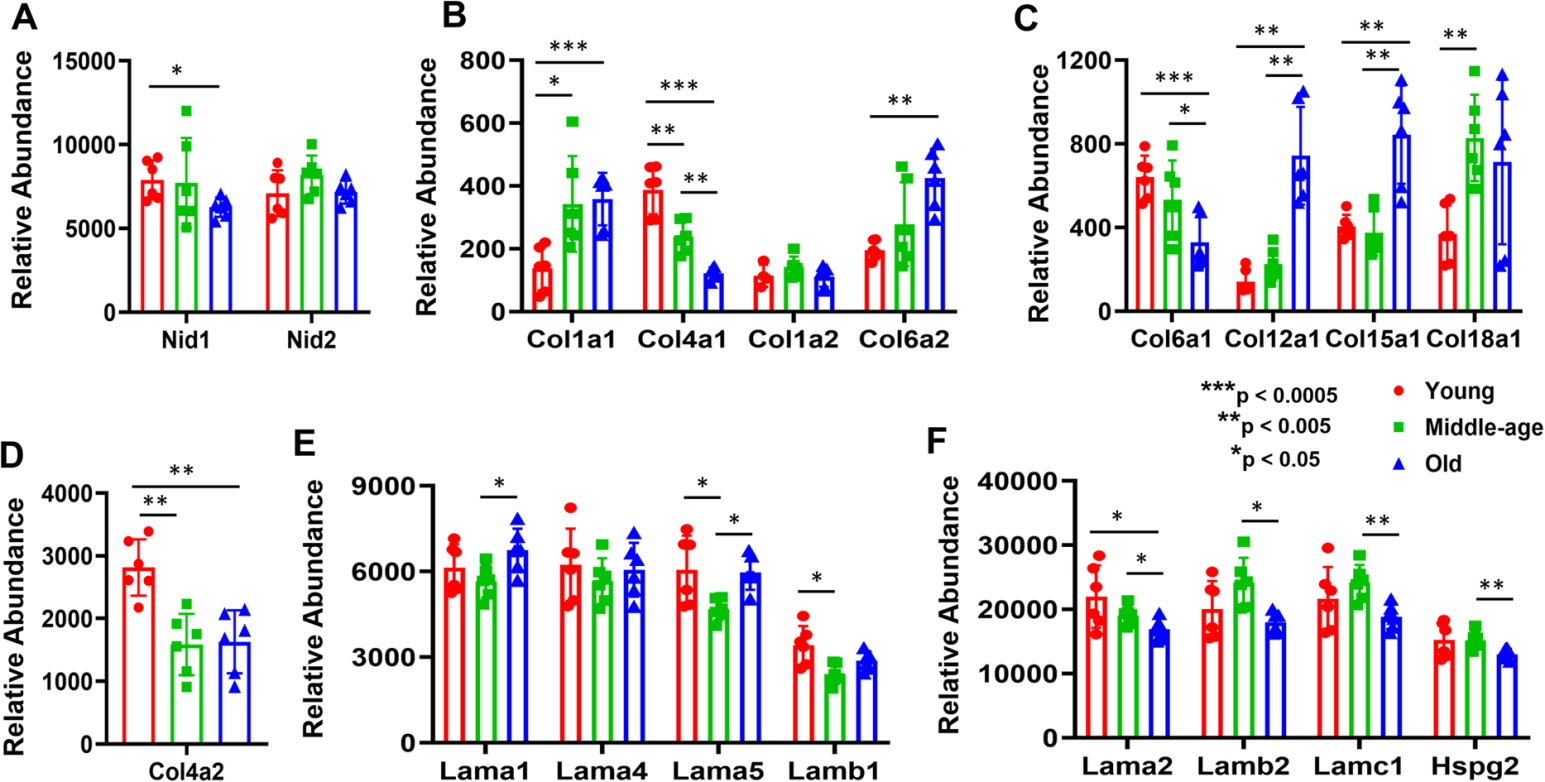
Altered expression of basement membrane proteins in mouse cortical MVs with aging (panels **A**–**F**). Proteins that exhibited between group differences are shown as colored bar graphs. Graphs show mean ± SD of relative abundance, with between group significant differences as indicated. Proteins presented in different panels passed the Shapiro–Wilk normality test followed by unpaired *t* test with Welch correction. Age-matched, three males and three females were included in each group (*n* = 6/group). Nid1 (NID1), nidogen-1; Nid2 (NID2), nidogen-2; Col1a1 (COL1A1), collagen alpha-1(I) chain; Col4a1 (COL4A1), collagen alpha-1(IV) chain; Col6a1 (COL6A1), collagen alpha-1(VI) chain; Col12a1 (COL12A1), collagen alpha-1(XII) chain; Col15a1 (COL15A1), collagen alpha-1(XV) chain; Col18a1 (COL18A1), collagen alpha-1(XVIII) chain; Col1a2 (COL1A2), collagen alpha-2(I) chain; Col4a2 (COL4A2), collagen alpha-2(IV) chain; Col6a2 (COL6A2), collagen alpha-2(VI) chain; Lama1 (LAMA1), laminin subunit alpha-1; Lama2 (LAMA2), laminin subunit alpha-2; Lama4 (LAMA4), laminin subunit alpha-4; Lama5 (LAMA5), laminin subunit alpha-5; Lamb1 (LAMB1), laminin subunit beta-1; Lamb2 (LAMB2), laminin subunit beta-2; Lamc1 (LAMC1), laminin subunit gamma 1; Hspg2 (HSPG2), heparan sulfate proteoglycan core protein

Basement membranes are composed of many structurally different components, and the composition of BMs varies according to anatomical location. For cortical MVs, the BM is composed of nidogen, collagen, and laminin components and perlecan (HSPG2) (Fig. 7). While a modest decrease in old mice compared with young mice occurred with NID1, expression of NID2 did not change (Fig. 7A). The collagen proteins showed a complex pattern which differed among the subtypes. The expression of COL4A1, COL6A1, and COL4A2 showed a progress decrease in middle-aged and old mice, whereas COL1A1, COL6A2, COL12A1, COL15A1, and COL18A1 showed increases either/and in middle-aged and old age compared with young mice (Fig. 7B–D). Laminin subtypes also showed a mixed response (Fig. 7E–F). Only LAMA5 and LAMB1 showed a reduction in middle-aged compared with young mice, whereas LAMA1 increased and LAMA2 decreased in old compared with middle-aged mice. For perlecan, a decrease in expression was seen in old compared with middle-aged mice (Fig. 7F).

## Discussion

**Fig. 8:**
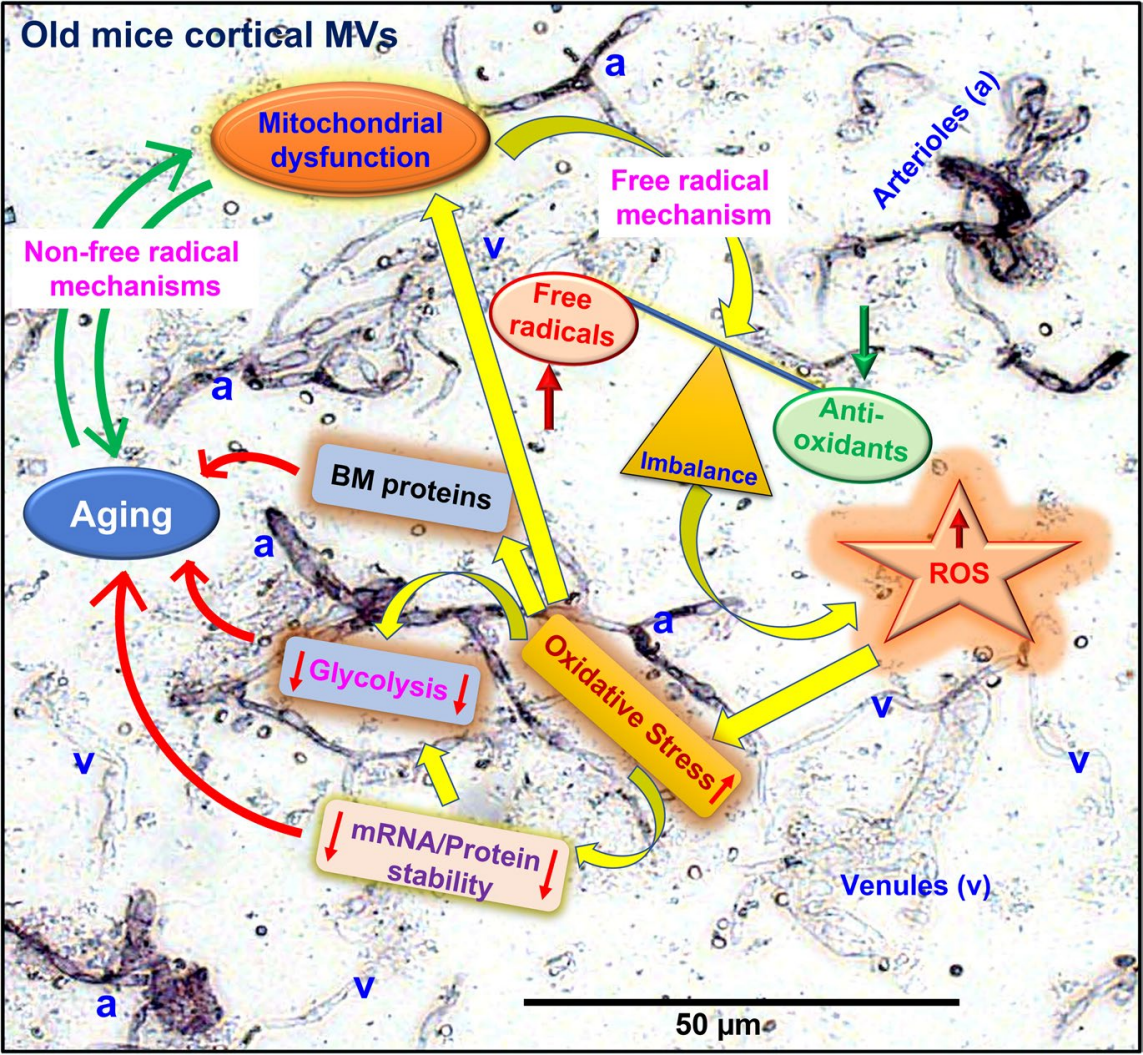
Putative events leading to stress and dysfunction of the brain MVs in aging. Brown, alkaline phosphate staining [ref 12, 13] in normal microscopy shows the isolated cortical MVs as a mixture of arterioles (a), capillaries, and venules (v). This study indicated that the reduction of ROS scavenging ability and subsequent increased oxidative damage, and decreased mRNA/protein stability, represent early, precipitating events leading to energy failure and basement membrane (BM) disruptions in the cerebral microvasculature of the aging brain

The major finding of this study is that significant changes occur in the protein composition of brain MVs of old mice, which increases the vulnerability of this cerebral vascular segment to ongoing damage and dysfunction, negatively affects cognition, and increases susceptibility to brain injury and disease. First, proteins regulating mRNA/protein stability are deranged starting in mid-life and continuing to old age. Thus, the length of time that intact mRNAs are available for translation is reduced, which leads to decreased protein synthesis, and those proteins that are synthesized will have a reduced lifespan in their original form. Second, important ROS scavenger levels are reduced with aging, corresponding with reports of increased ROS availability in old age. Third, enzyme levels involved in glycolysis and many components of mitochondrial complexes I–V are reduced, which will decrease the ability of MVs to provide the necessary ATP response to stress and injury. Fourth, the BM components undergo aging changes, which may lead to inappropriate alterations in MV structural integrity. We propose that reduced ROS scavenging ability coupled with subsequent increased oxidative damage and mRNA/protein stability are early, precipitating events leading to energy failure and BM disruption (Fig. 8—schematic). In addition, the results indicate that detrimental effects of normal aging occur as early as 12–14 months in mice and thus provide support for the view that therapies, especially in vulnerable individuals, should begin in mid-life.

### ROS scavenger proteins during aging

A dominant theory in the aging literature focuses on the accumulation of damaging effects of ROS on cells which could arise via enhanced ROS production, decreased antioxidant systems, or a combination of both [17, 18]. Using two photon microscopies, Han et al. [19] detected increased levels of ROS in the cerebral vessels of old mice. However, despite overwhelming evidence supporting the idea that ROS are produced and can manifest damage in cells, a causal link between ROS and normal aging has not been clearly established. Mitochondria are major source of ROS in aging brain, but other important contributors include enzymes within the plasma membrane, NADPH oxidases (NOXs), lipid metabolism, and various cytosolic enzymes such as cyclooxygenases. In the vasculature, NOX enzymes are a substantial source of ROS, and are key players in mediating redox signaling under physiological and pathophysiological conditions including age-associated diseases [20–26]. However, the contribution of NOX enzymes as a source of oxidant stress during normal aging process has not been clearly established. While we did not measure ROS production or the source of ROS in MVs, we intend to do so in future studies. Nonetheless, reduced ROS scavenging systems during aging compromise the brain microvasculature. There are studies supporting the concept that a decrease in antioxidant defenses appears with aging [27–29] which support our findings. Postmortem studies of different human brain regions uncovered a gradual age-related decrease in SOD and CAT as well as GSH reductase activity, mainly in the hippocampus and frontal cortex [30]. In line with these observations, in vivo monitoring of GSH content in the human brain from healthy subjects exhibited a steady decrease in this antioxidant enzyme in old compared with young individuals [29]. Studies in old rat brains revealed a decrease in SOD2 activity compared with young rat brains [31]. In this regard, we found that the relative abundances of SOD1, SOD2, CAT, and TXN1 were significantly decreased in cortical MVs of old mice (Fig. 1A–D). The decrease in the relative abundance of CAT and TXN1 in middle age indicates that the potential for increased oxidative stress becomes manifest relatively early in mice MVs. In addition to the primary antioxidants, many secondary antioxidants such as GPX1 form redox cycles that offer necessary cofactors for primary antioxidants and can also function as direct scavengers of ROS [32–35]. Although the expression of GSS and GPX1 was not decreased with aging, the redox cycle proteins such as GGT1 and GSTK1 were also significantly decreased in old mice MVs (Fig. 1E–H). These enzymatic and nonenzymatic antioxidant systems are essential to ensure the health of cells by maintaining the optimal redox balance as well as decrease or avoid cellular damage caused by ROS [36–38].

### mRNA/protein stability during aging

Messenger RNA turnover mechanisms regulate the lifetime of cytoplasmic mRNAs as a means of controlling gene expression under both normal and stress conditions, whereas the impact of mRNA turnover on aging and age-related disorders has recently become apparent [39]. Age-dependent changes in mRNA decay are currently unknown; however, some mechanistic pathways are beginning to emerge [40]. Several age-related neurodegenerative disorders are associated with deficiencies in RNA-binding protein function and play regulatory roles in longevity [41–44]. Many proteins, including cytoplasmic poly(A)-binding protein 1 (PABPC1), bind the poly(A) tail of mRNA, including that of its own transcript, and regulate mRNA metabolism processes and mRNA stability [45–47]. We observed that PABPC1 expression was significantly decreased in cortical MVs with aging (Fig. 2A). On the other hand, the mRNA processing bodies (also known as P-bodies) are mainly involved in translational repression and mRNA decapping and degradation. The P-body components include the decapping enhancers LSM1–7, and the 5΄ to 3΄ exonuclease XRN1 or XRN2 that regulate the decapping or degradation rate of mRNAs [48–50]. In the 3΄ to 5΄ decay pathway, mRNAs are degraded in this direction by the SKI RNA helicase complex [51, 52]. We observed that the expression of PAPD5, XRN2, SKIV2l2, and LSM7 involved in the mRNA degradation or pre-mRNA splicing critical for longevity [53, 54] were upregulated in MVs of old mice (Fig. 2D–G). Alternative RNA splicing is accomplished by large ribonucleoprotein complexes, known as spliceosomes. Several RNA binding proteins act as splicing regulators to expedite or inhibit splice site recognition by spliceosome components [55]. A gene ontology analysis in both human and mouse reported that changes in pathways such as mRNA binding, RNA processing, and RNA splicing are strongly associated with age [56, 57]. Age‐related splicing fluctuations in the human brain affect pathways such as sugar metabolism and DNA repair [58], both pertinent to aging [59, 60]. Recently, Ubaida-Mohien et al. [61] reported that spliceosomal proteins were increased by ~ 15% between the ages of 20 and 87 years, and they propose that changes in the splicing machinery enable muscle cells to respond to a rise in damage with human aging. Systematic changes in the splicing machinery with older age were also suggested by epidemiological studies [62], transcriptomic analyses of skeletal muscle biopsies [63, 64], and human peripheral blood leukocytes [57] of young and old individuals. During or after translation, proteins adapt their structures in a process called folding. Generally, folding is accelerated by chaperones and associated protein activity. Decreased chaperone capacity with age has been shown in numerous studies [65–67]. We observed that HSPA9 and HSPD1 were involved in protein folding and were decreased more significantly in MVs of middle-aged and old than in young mice (Fig. 2B–C). The signaling process concerning whether to degrade a protein is affected in part by the availability of ATP in the cell. Decline of cellular energetics with age and disruption of fatty acid and glucose metabolism decreases the amount of available ATP and changes chaperone activity, leading to the accumulation of damaged proteins [68, 69].

### Anaerobic glycolysis during aging

Glycolysis is crucial for energy production in the developing brain, whereas OXPHOS becomes more dominant in the mature brain [70]. We are unaware of any studies which have considered the effects on glycolysis of aging brain cortical MVs. Recently, brain glycolysis has been recognized as a process not only involved in hypoxic conditions, but also as a critical pathway affecting signal transduction, synaptic activity, learning, and brain development [70–73]. In normoxic conditions, glycolysis can promptly increase intracellular ATP levels with the changing demands of a cell for activation, proliferation, secretion, migration, and apoptosis [74–76]. Moreover, the metabolic control of angiogenesis or barriergenesis may be provided by glycolytic activity of the neurovascular unit cells [77]. The ATP required by neurons [78], and microglia [79] is predominantly generated within mitochondria by OXPHOS. In contrast, energy requirements of astrocytes [80] and oligodendrocytes [81] are predominantly met by glycolysis. In the cerebral vasculature, glycolysis promotes vessel branching [82, 83], and whereas endothelial migration is associated with angiogenic events [82], the suppression of glycolysis results in impairment of angiogenesis [84, 85]. During angiogenesis, the developing capillaries are more permeable than established vessels [86]. However, there appears to be an age-related decrease in the capacity for cerebral angiogenesis [87, 88]. Several vascular density studies in aging rats reported decreases in capillary number, length, volume, and vascular density in hippocampus, cortex, white matter, and brain stem regions [89–96]. Similarly, in human aging, decrease in capillary/vascular density in cortex and other brain areas is also reported in several studies [3–5, 97]. During aging, the human brain experiences normal changes including a global decline in glucose metabolism, oxygen consumption, and cerebral blood flow [71, 98–100]. Recently, Goyal et al. (2017) reported that average aerobic glycolysis gradually decreases with age, approaching zero at the whole-brain level close to the age of 60 [98]. These conclusions are strongly supported by a prior quantitative study in cognitively normal active young and older adults [101]. In healthy aging, brain glucose metabolism decreases mainly in the frontal cortex, whereas in AD and other neurodegenerative diseases, the parietal lobe and precuneus are the most significantly affected [102]. Our present study indicates that except for TPI, the expressions of all other glycolytic enzymes were significantly decreased with aging in mice cortical MVs (Fig. 3). Inhibition of the “house-keeping” glycolytic enzyme, GAPDH, by nitric oxide results in higher BBB permeability and barrier dysfunction [103]; the same effect is provoked by overproduction of ROS in endothelial mitochondria [104]. The aging-related decrease of anaerobic glycolysis may also indicate a loss of neuroprotection against oxidative stress via the pentose phosphate pathway, increasing the risk for oxidative damage. The aging-related loss of glycolysis in the absence of an amyloid or neurologically distinct brain pathology suggests an underlying physiological change that harbors poor outcomes to the aging brain. Decreased anaerobic glycolysis might provide a template for the onset of more severe brain energetic deficits in neurodegenerative diseases [98, 105–108].

### Mitochondrial proteins during aging

The mitochondrial theory of aging hypothesizes that mitochondria are the essential component in control of aging. Thus, the dynamic interactions between mitochondrial and glycolytic activity in endothelial cells is essential for maintaining endothelial layer integrity. Our proteomics study indicates that the number of significant mitochondrial proteins in cortical MVs sequentially decreased from young to middle-aged to old mice. Moreover, a significantly higher number of mitochondrial proteins were expressed in young male (~ 67%) than female (~ 33%) MVs (Supplementary Table 2). However, this trend reversed in middle-aged mice MVs, and surprisingly, only six mitochondrial proteins were significantly differentially expressed in old male MVs (Supplementary Table 2). Increasing evidence, however, indicates a role for changes in mitochondrial function as a probable central regulator of the aging process. Abnormally rounded mitochondria [109] decreases in mitochondrial number [110] as well as mitochondrial DNA (mtDNA), and copy number decreases [111–113] are significantly linked with aging. We have previously shown that mitochondrial respiration is reduced in old mice [14]. Mitochondrial complex I (MC-I) is thought to be a site of impairment since more subunits are encoded by mitochondrial rather than nuclear DNA. Due to its proximity to ROS produced by mitochondria, mtDNA is considered to be more susceptible to oxidative damage [114–116]. MC-I is often cited as the most likely site of an electron transport chain impairment [114, 117–119]. A human, postmortem study of different brain regions revealed a progressive age-related decrease in MC-I activity, mainly in the hippocampus and frontal cortex [30]. In nonhuman primates, ATP synthesis capacity and pyruvate dehydrogenase activity were decreased in the putamen of old compared with young animals [120]. In mice [121, 122] and rats [119], the functional impairment of MC-I activity was also evident in the brain of old compared with young animals. A strong positive correlation was demonstrated between decreased MC-I functionality and increased ROS production [119]. Antioxidant defensive failures were coupled with decreases in MC-I, MC-IV, and ATP synthase activity, leading to a reduction in ATP production [123, 124]. Our study supports the concept that aging influences the expression of proteins in MC-I, MC-II, and MC-III (Fig. 4) in mouse cortical MVs. Moreover, the expression of proteins involved in MC-IV and MC-V (ATP synthase) was also significantly decreased in old mice MVs (Fig. 5 and 6).

### Basement membrane during aging

The BM, an extracellular matrix, provides additional structural support to the BBB. The BM is composed of collagen, which provides structural integrity and flexibility. Laminin combines with type IV collagen to provide a mesh like framework for binding other proteins such as nidogen, which stabilize the BM as it binds to and bridges laminin, collagen, and perlecan, as well as provides for charge-dependent filtration and signal transduction. Type IV collagen subunits, classical fibrillar collagens, appear to be the predominant subtype in the MV BM, but the other subtypes are important in providing structural support. BM composition and structure is tissue‐specific and dynamic [125] and undergoes compositional and structural changes with aging [126, 127], including the cerebrovascular BM [128]. Our results indicate that considerable rearrangements of the BM, especially in collagen subtypes, occur during aging. For example, the relative abundance of type IV collagen subtypes shows a substantial decrease starting in middle age and continuing in old age, while collagen subtype 1A1, a fibrillar collagen, shows increases with aging. Additionally, BM zone collagens such as collagens XV and XVIII, and FACIT-like/short chain collagens such as collagen 12A1, show increases with aging. Thus, a shift from longer to shorter collagens appears to occur with aging. Collagen 6, another shorter collagen, appears to undergo subunit substitution as collagen 6A1 decreases, while 6A2 increases. A decrease in many but not all of the laminin subtypes and perlecan is consistent with a decrease in collagen 4. We speculate that the BM in old mice is not as tightly woven, is more permeable, and is less flexible than in younger mice MVs.

### Blood–brain barrier disruption in the aging brain

BBB breakdown is an emerging biomarker in normal aging [129, 130]. Increases in oxidative stress, and the decreased stability of mRNA and/or proteins, declining ATP production, and changing BM proteins lead to altered BBB integrity in aging brain. Ungvari and colleagues reported that aging aggravated obesity-induced brain microvascular damage and BBB disruption in the mouse hippocampus [131]. In aging, oxidative stress induces cerebral endothelial cells to produce TNF-α that trigger the degradation of the BM, and TJ-proteins, which, in turn, results in BBB disruption and an increase in BBB permeability [132–134]. Inhibition of the glycolytic enzyme, GAPDH, results in higher BBB permeability and barrier dysfunction [103]; the same effect is provoked by overproduction of ROS in endothelial mitochondria [104]. In our discovery-based proteomics study, we also identified that expression of TJ-proteins is significantly decreased in cortical MVs of old mice indicating the probable disruption of BBB in aging brain (unpublished data).

## Conclusions

The results of our study support the concept that reduced ROS scavenging ability and mRNA/protein stability are early, precipitating events leading to energy failure and BM disruptions, and which subsequently lead to adverse changes in proteins supporting ATP production by glycolysis and OXPHOS and structural integrity of the BBB-supporting BM. Thus, the results indicate that detrimental effects of normal aging occur as early as 12–14 months in mice and thereby provide support for the view that therapies, especially in vulnerable individuals, should begin in mid-life. While we are not yet able to define with certainty the specific critical events that lead to a compromised and vulnerable brain microvasculature, our results indicate likely targets for further investigation which we expect will lead to novel therapies to protect not only the microvasculature but also the brain parenchyma.

## Supplementary Information

ESM 1 (PNG 41423 kb)

High resolution image (TIF 4821 kb)

ESM 2 (PNG 87818 kb)

High resolution image (TIF 4984 kb)

ESM 3 (PNG 25560 kb)

High resolution image (TIF 1421 kb)

ESM 4 (PNG 61829 kb)

High resolution image (TIF 3465 kb)

ESM 5 (PNG 1126517 kb)

High resolution image (TIF 6765 kb)

ESM 6 (PNG 1212506 kb)

High resolution image (TIF 8606 kb)

ESM 7 (PNG 2769 kb)

High resolution image (TIF 228 kb)

ESM 8 (PNG 3460 kb)

High resolution image (TIF 270 kb)
