# Supplementary figures and images for: Effects of aging on protein expression in mice brain microvessels: ROS scavengers, mRNA/protein stability, glycolytic enzymes, mitochondrial complexes, and basement membrane components
Source: GeroScience. 2021 Oct 28;44(1):371–88. doi: 10.1007/s11357-021-00468-1 (PMC8811117; doi:10.1007/s11357-021-00468-1)

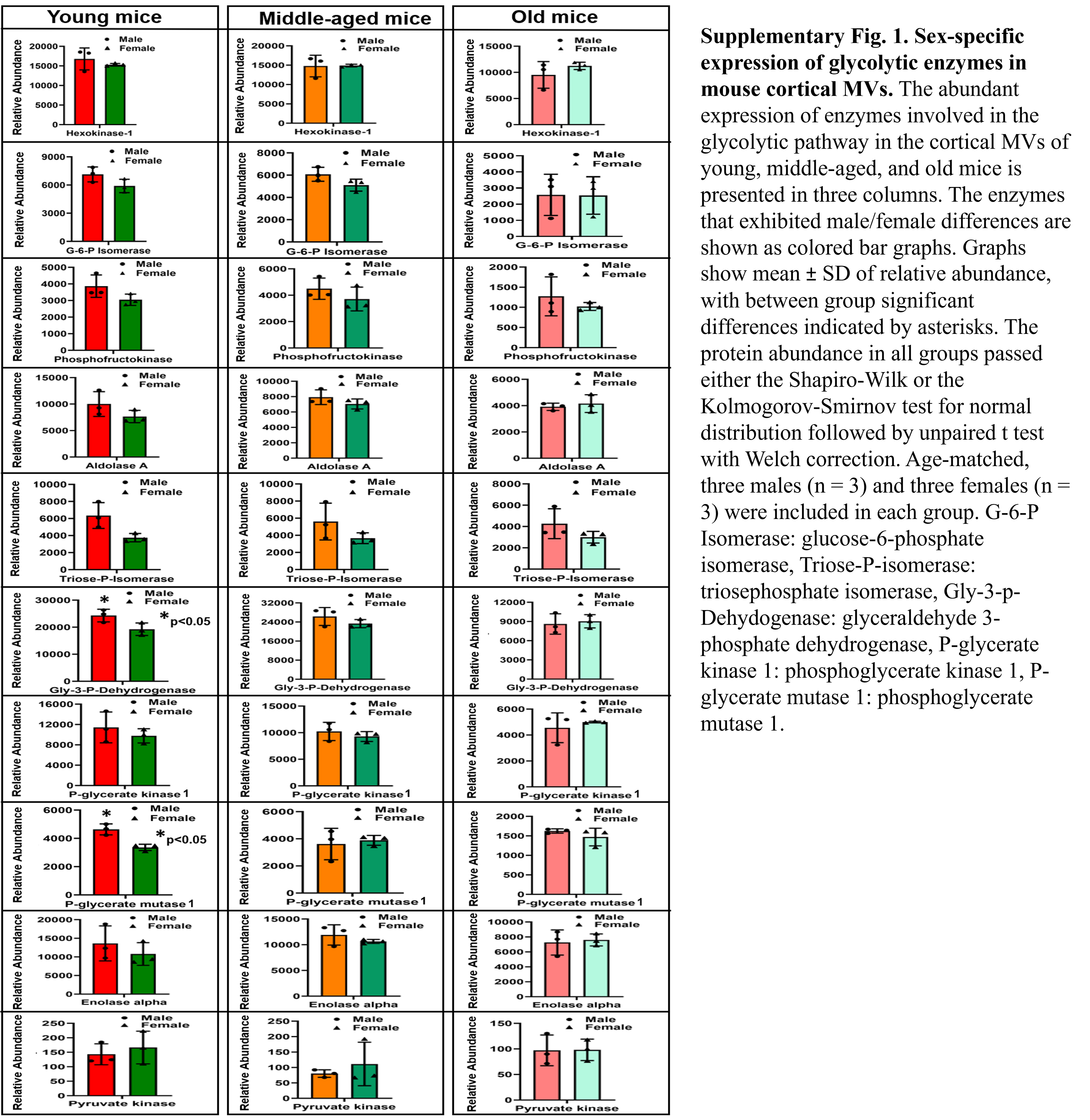

Supplement: Supplementary file 1 — (PNG 41423 kb) [file 11357_2021_468_Fig9_ESM.png]

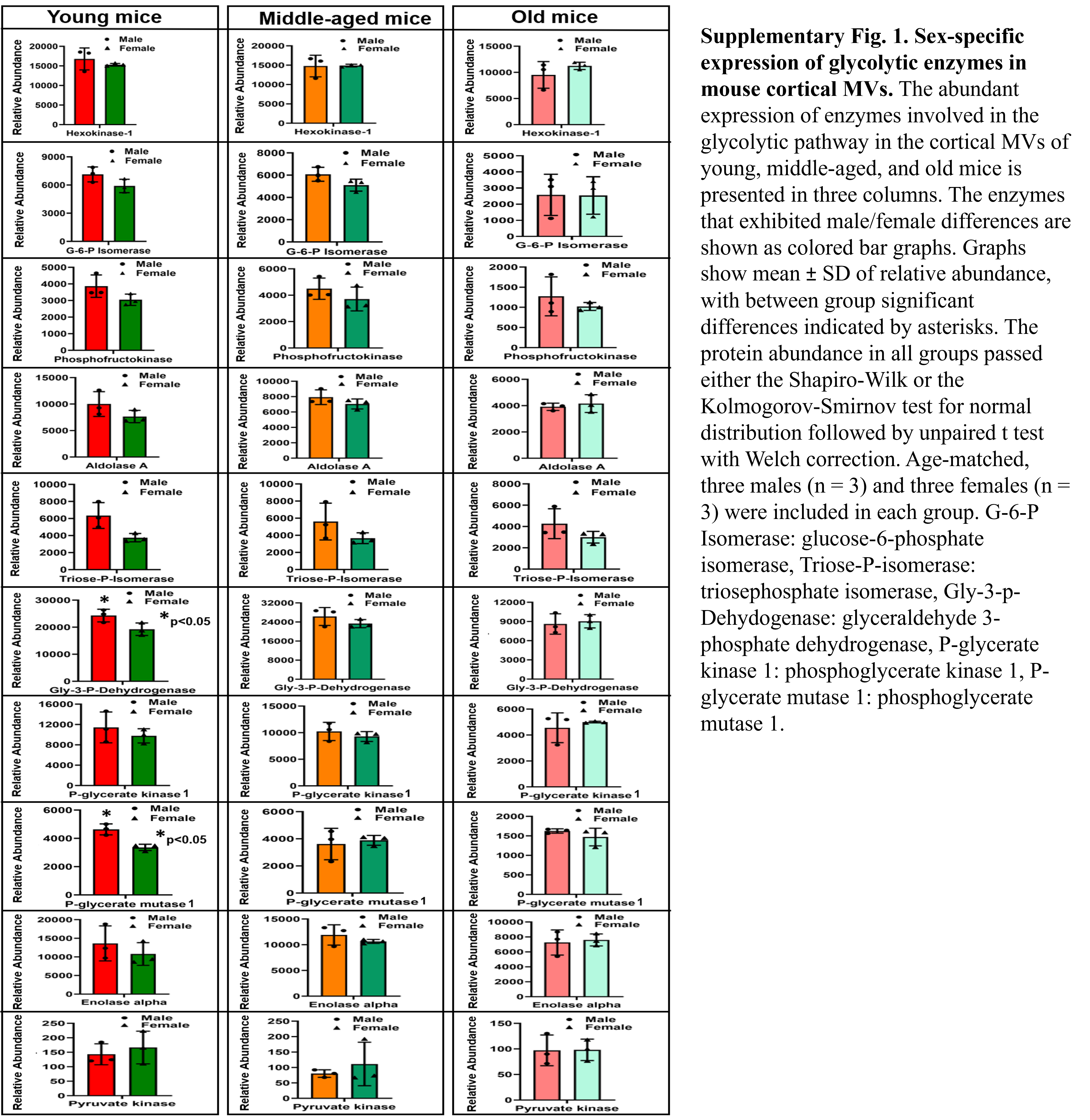

Supplement: Supplementary file 2 — High resolution image (TIF 4821 kb) [file 11357_2021_468_MOESM1_ESM.tif]

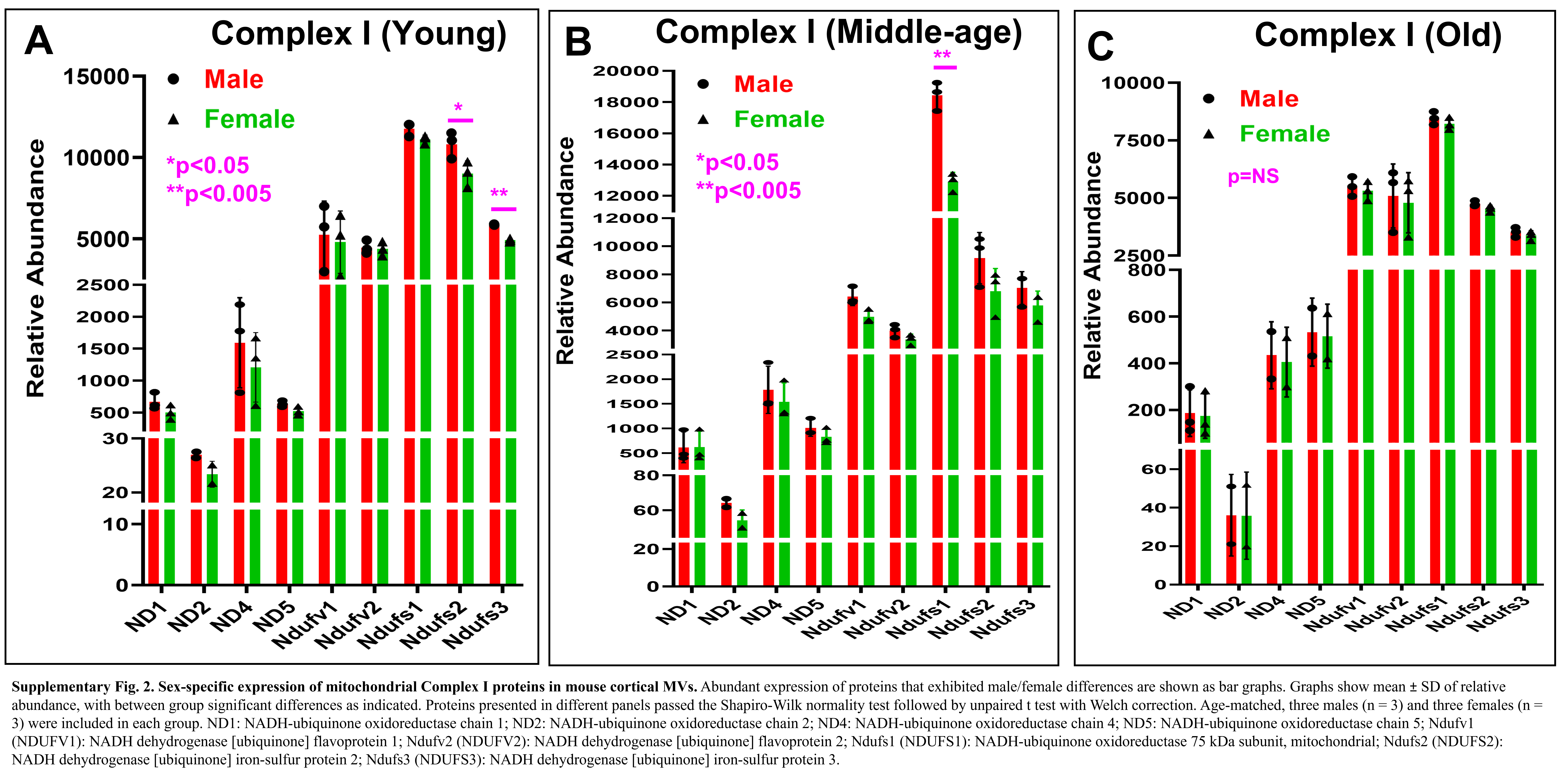

Supplement: Supplementary file 4 — High resolution image (TIF 4984 kb) [file 11357_2021_468_MOESM2_ESM.tif]

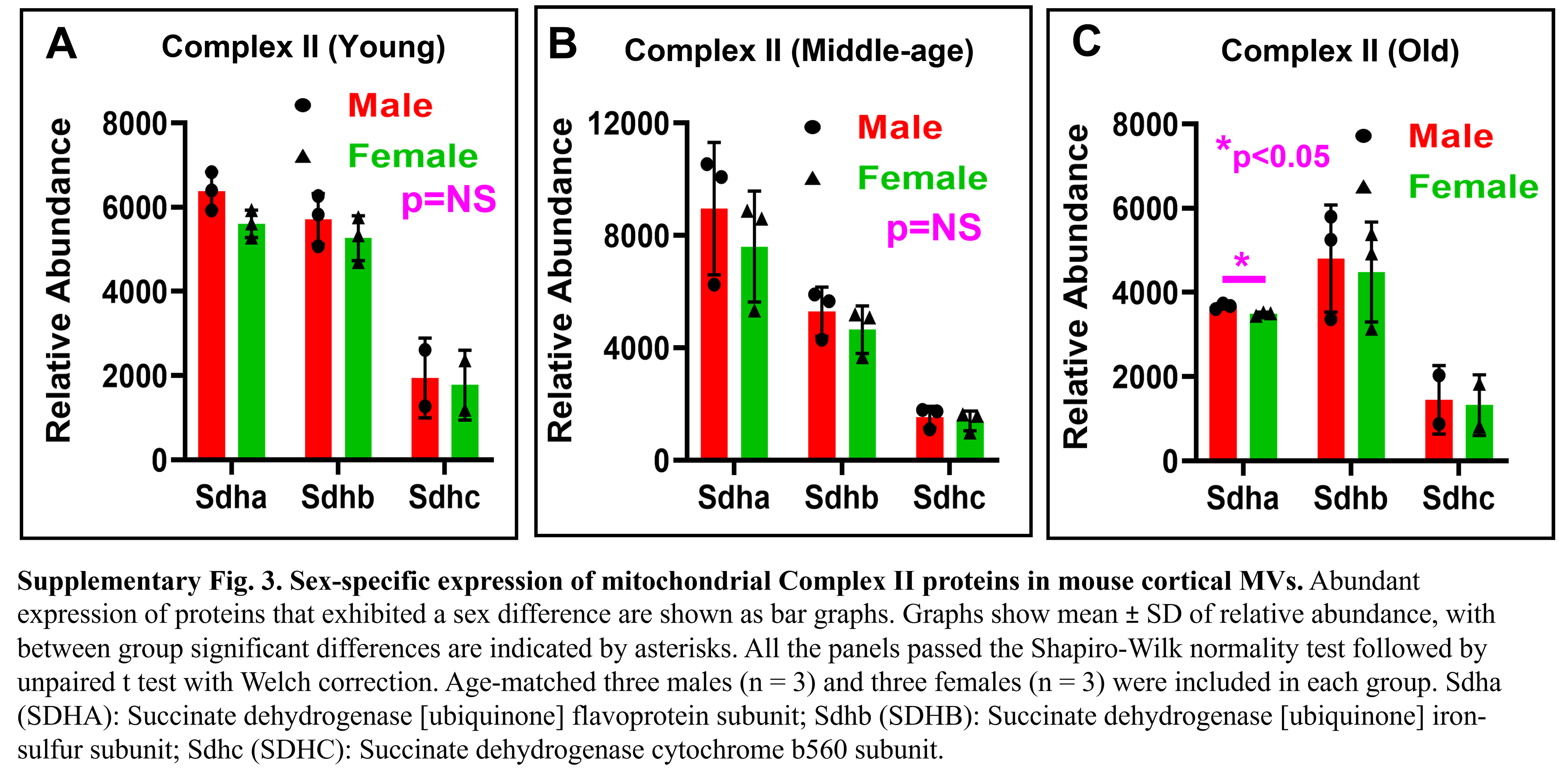

Supplement: Supplementary file 5 — (PNG 25560 kb) [file 11357_2021_468_Fig11_ESM.png]

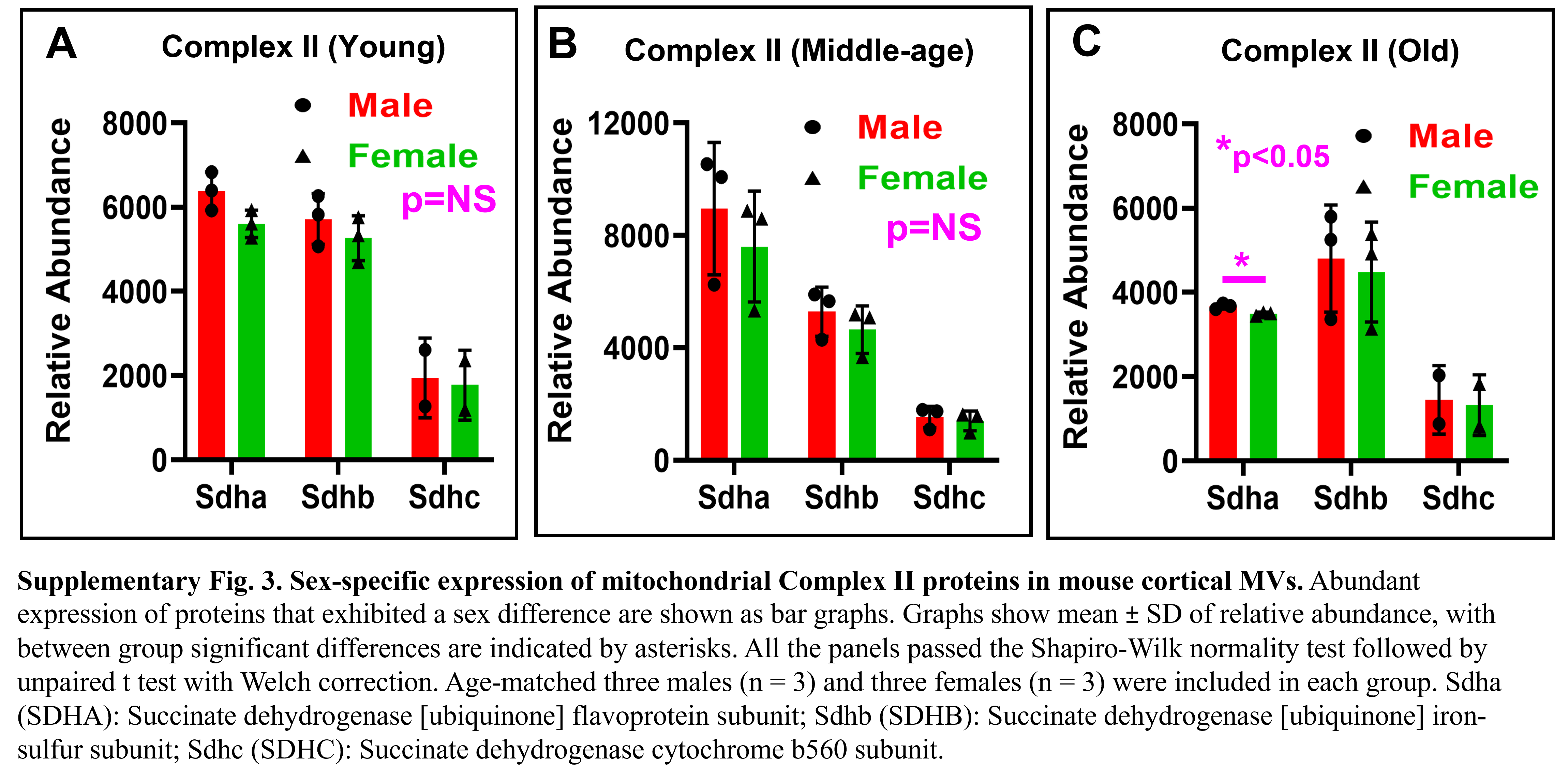

Supplement: Supplementary file 6 — High resolution image (TIF 1421 kb) [file 11357_2021_468_MOESM3_ESM.tif]

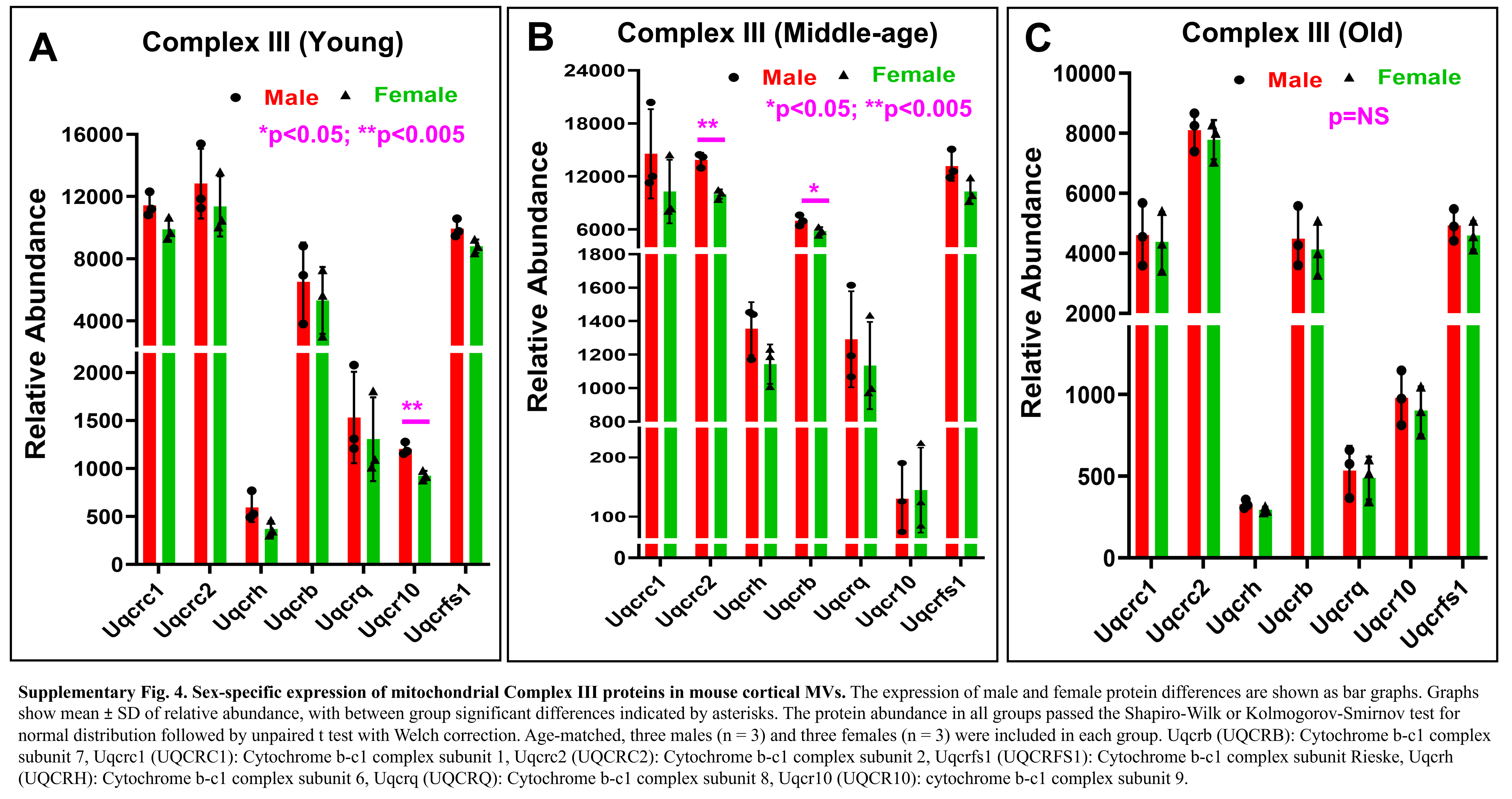

Supplement: Supplementary file 7 — (PNG 61829 kb) [file 11357_2021_468_Fig12_ESM.png]

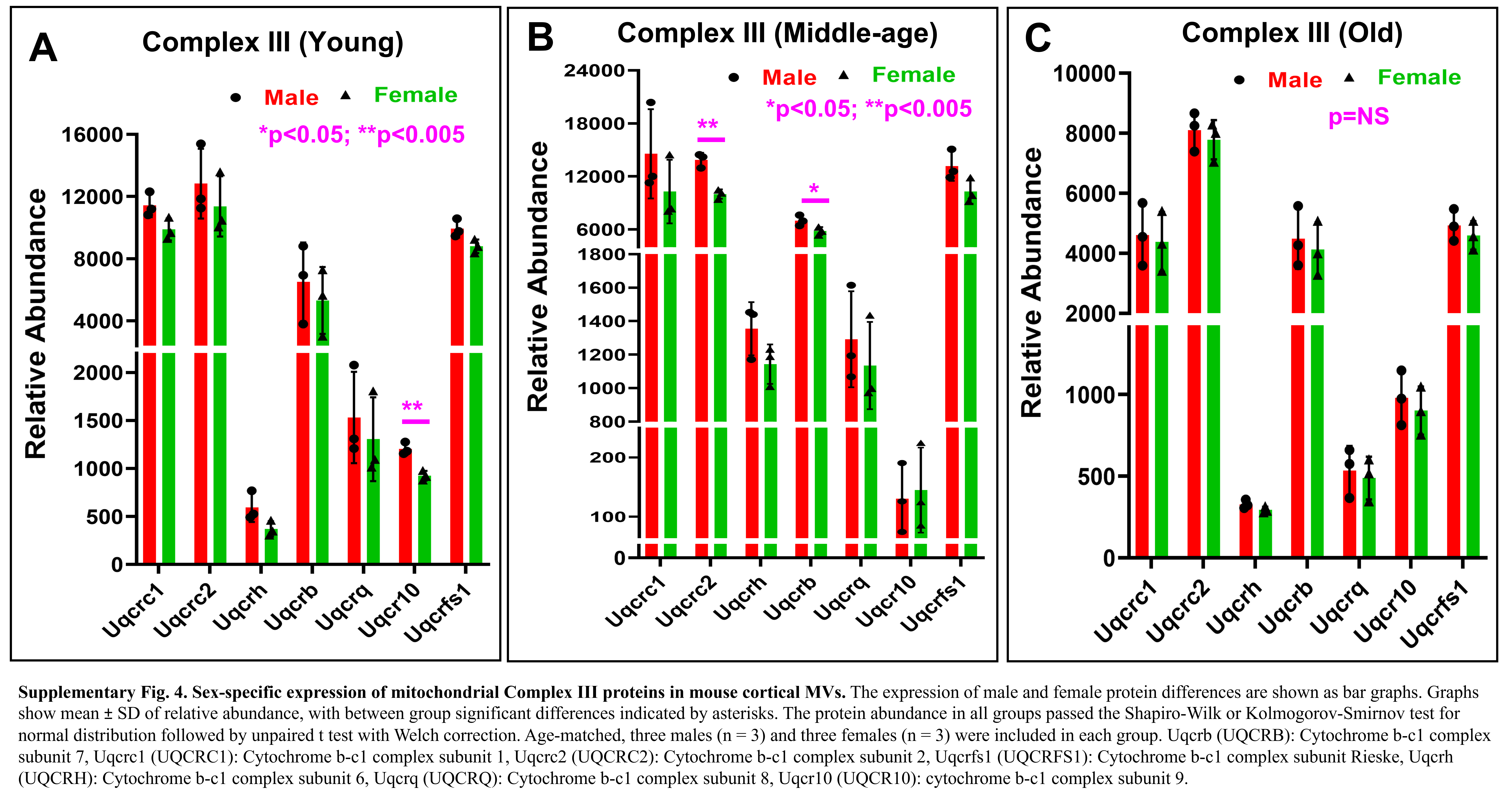

Supplement: Supplementary file 8 — High resolution image (TIF 3465 kb) [file 11357_2021_468_MOESM4_ESM.tif]

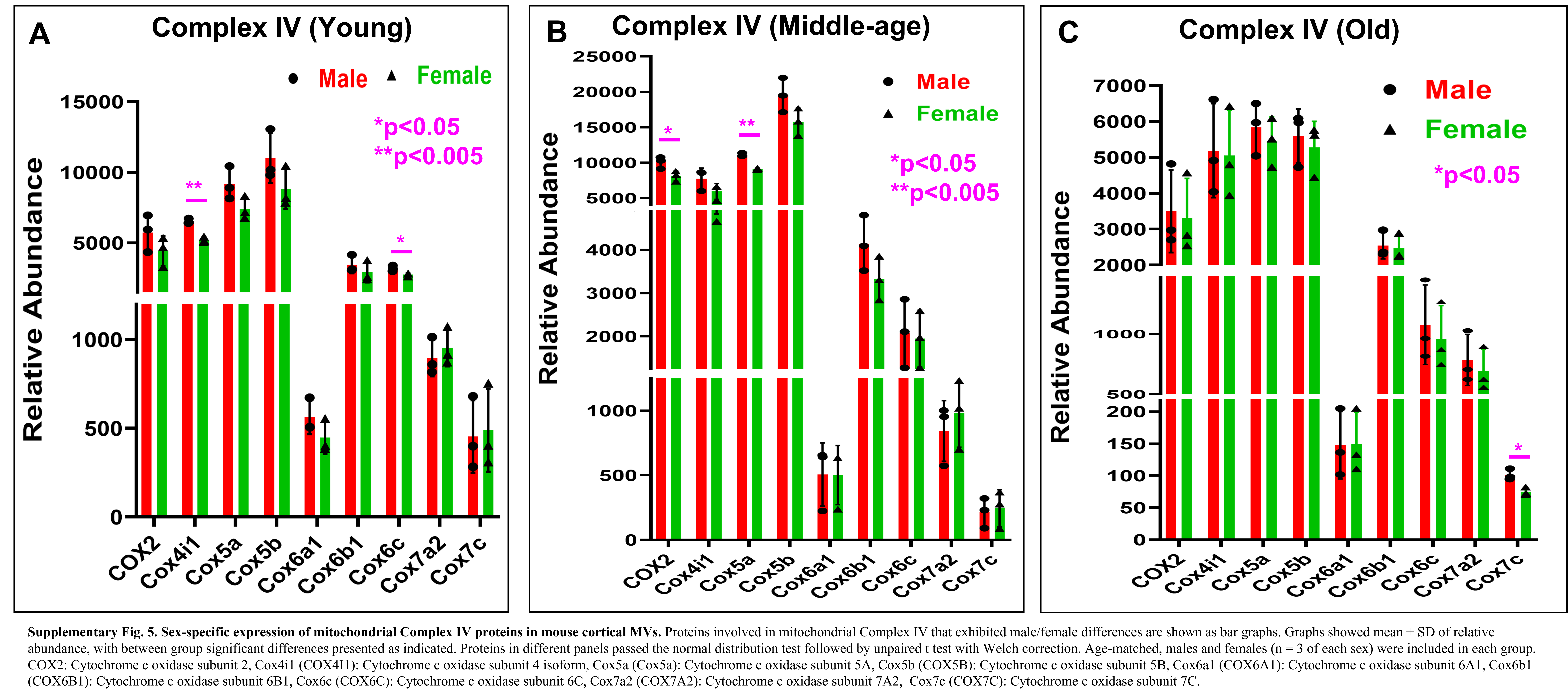

Supplement: Supplementary file 9 — (PNG 1126517 kb) [file 11357_2021_468_Fig13_ESM.png]

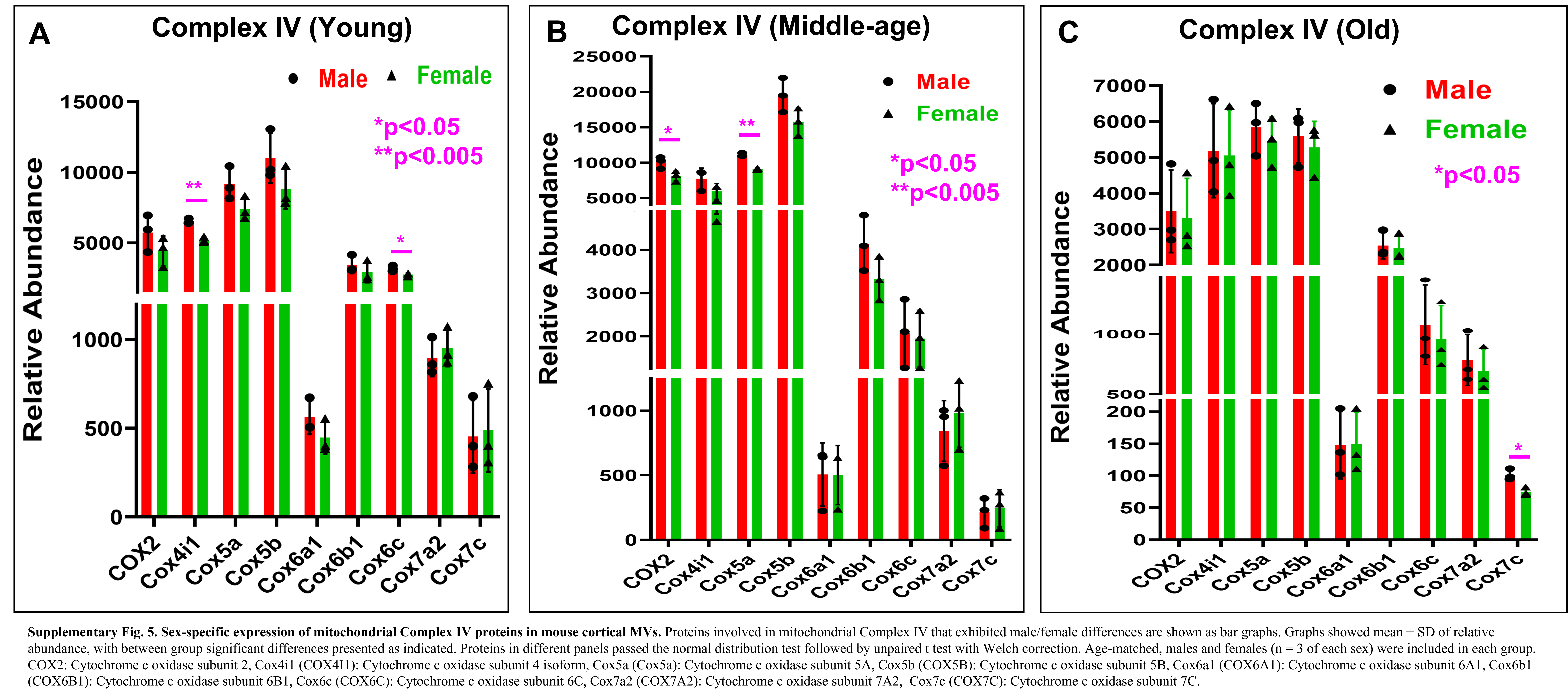

Supplement: Supplementary file 10 — High resolution image (TIF 6765 kb) [file 11357_2021_468_MOESM5_ESM.tif]

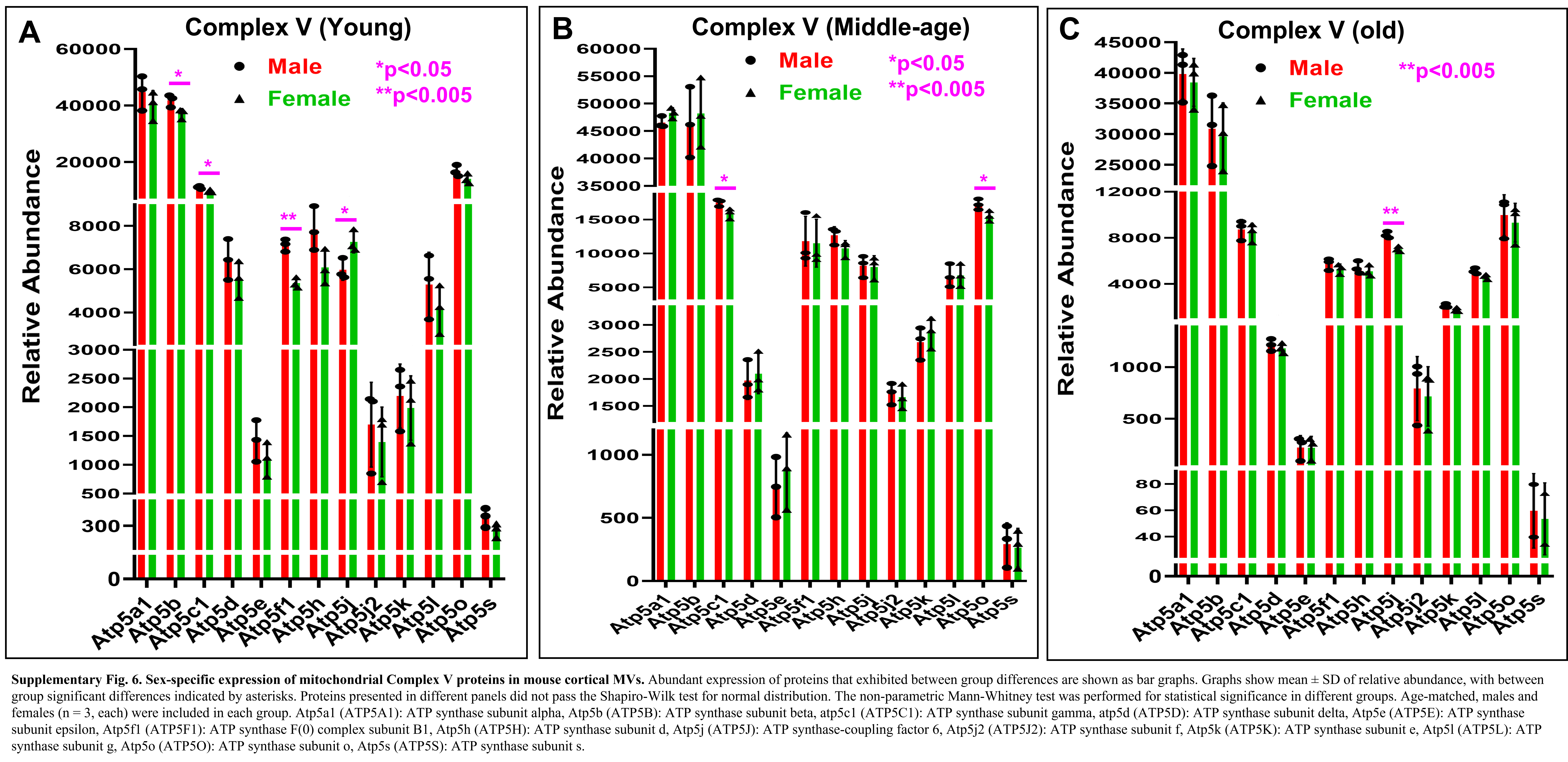

Supplement: Supplementary file 11 — (PNG 1212506 kb) [file 11357_2021_468_Fig14_ESM.png]

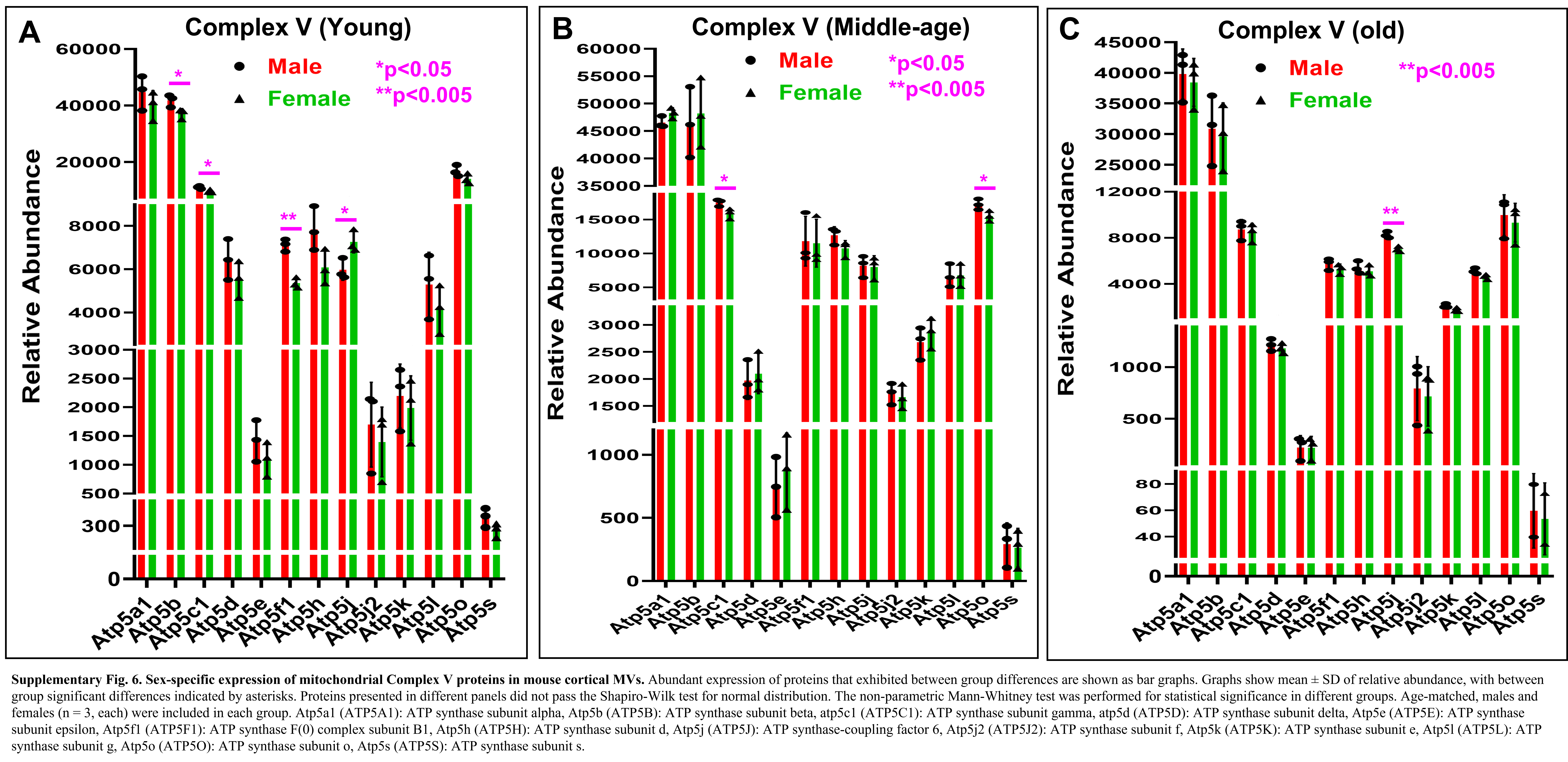

Supplement: Supplementary file 12 — High resolution image (TIF 8606 kb) [file 11357_2021_468_MOESM6_ESM.tif]

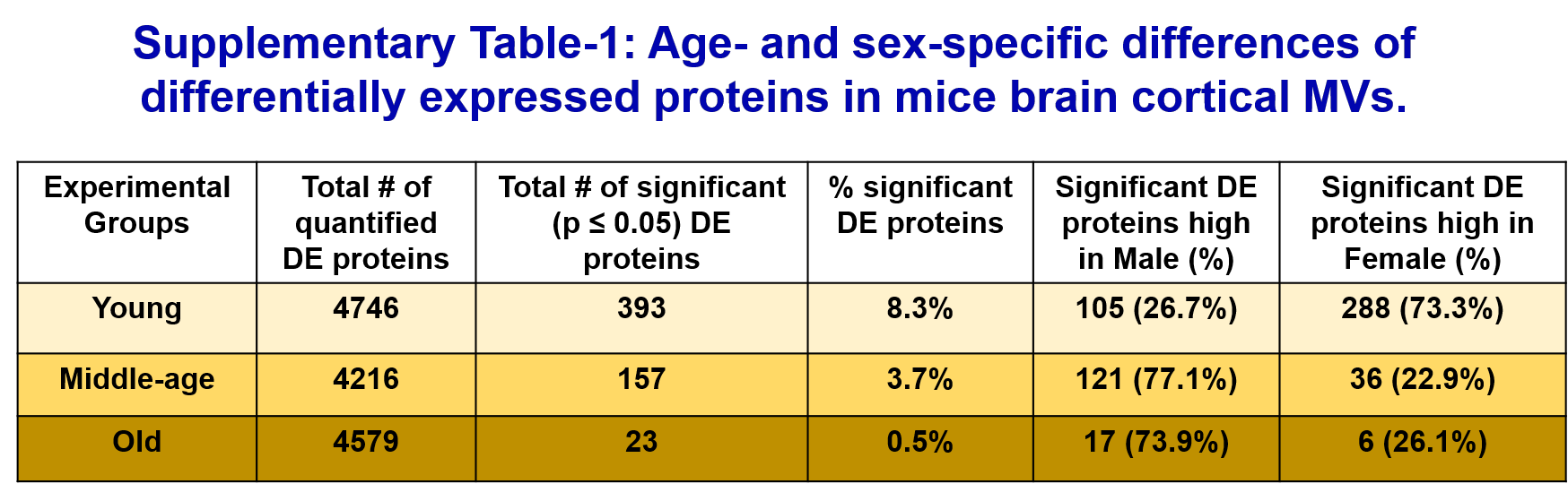

Supplement: Supplementary file 13 — (PNG 2769 kb) [file 11357_2021_468_Fig15_ESM.png]

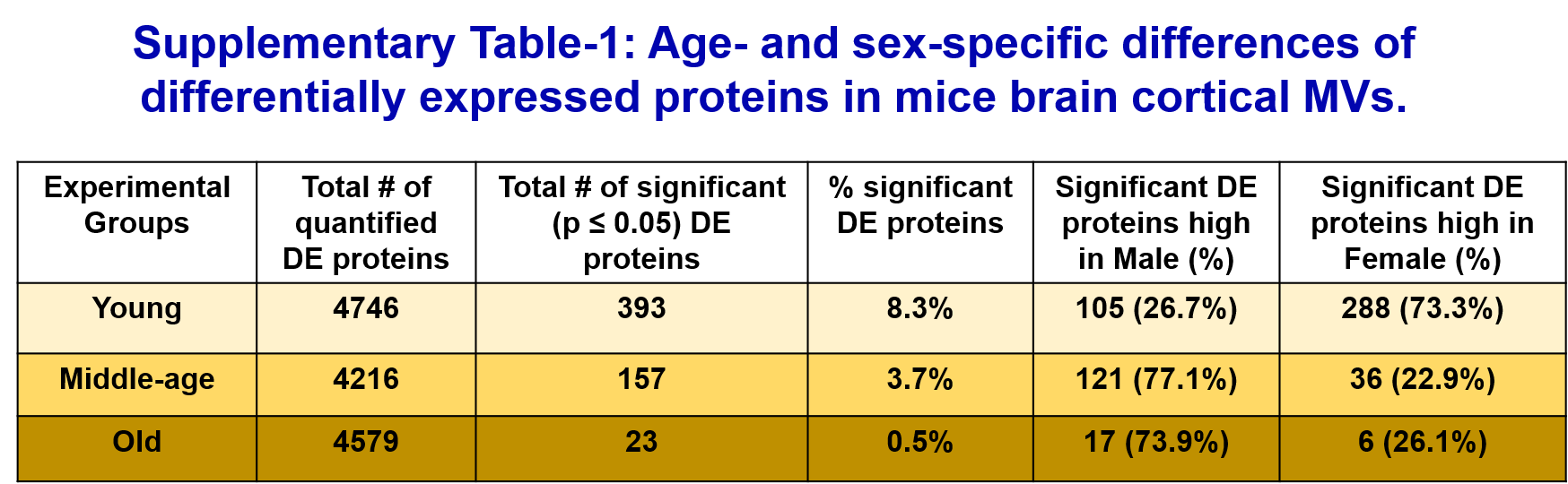

Supplement: Supplementary file 14 — High resolution image (TIF 228 kb) [file 11357_2021_468_MOESM7_ESM.tif]

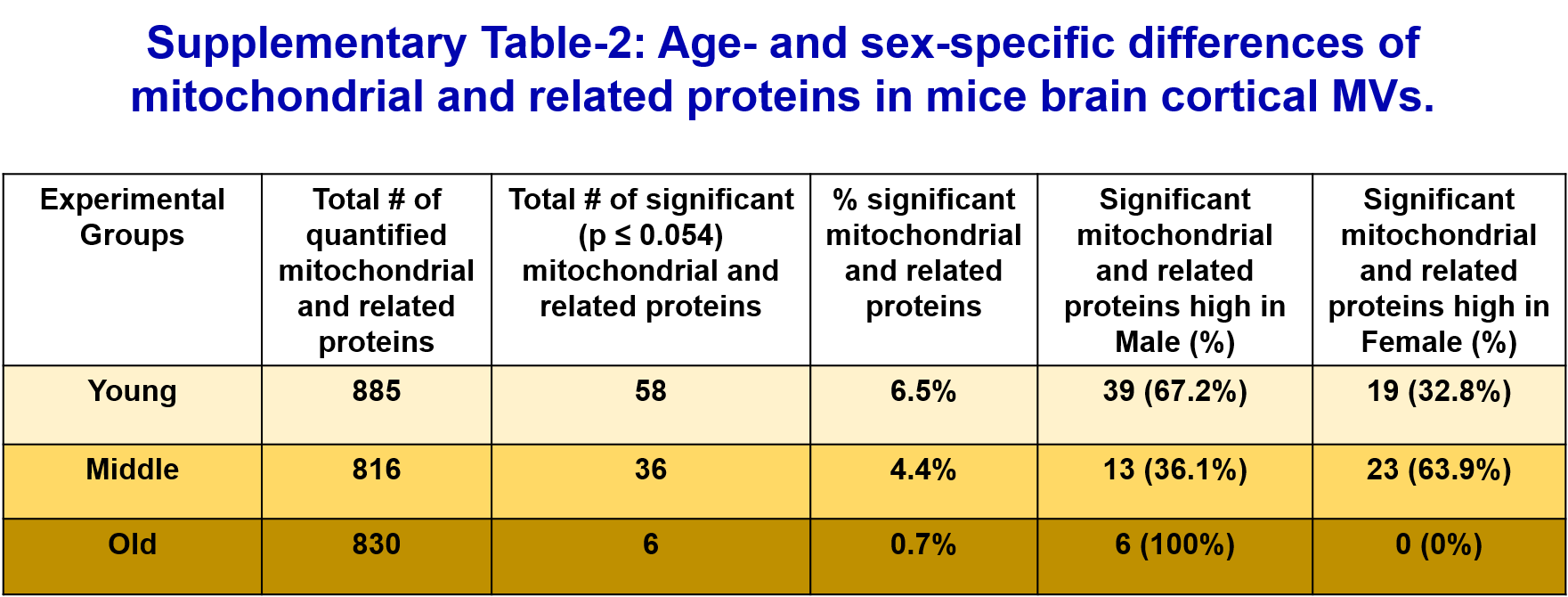

Supplement: Supplementary file 15 — (PNG 3460 kb) [file 11357_2021_468_Fig16_ESM.png]

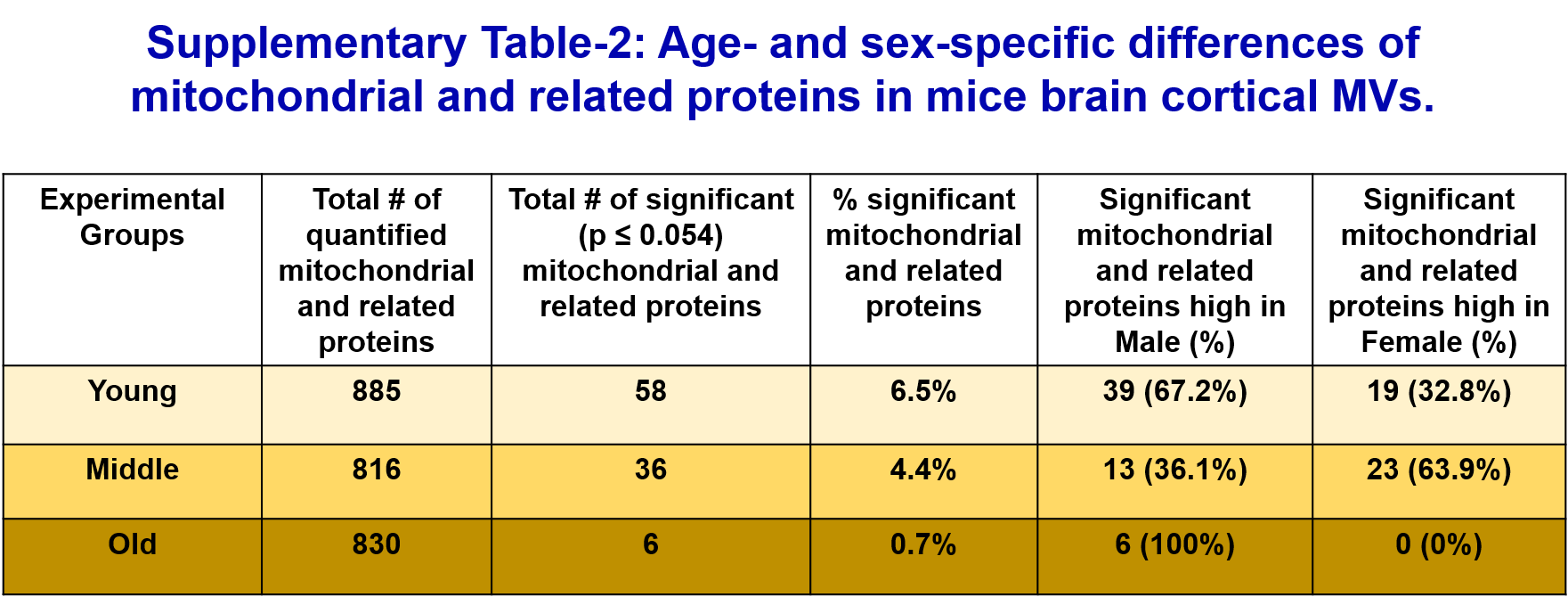

Supplement: Supplementary file 16 — High resolution image (TIF 270 kb) [file 11357_2021_468_MOESM8_ESM.tif]
